# Differential gene expression and transport functionality in the bundle sheath versus mesophyll – a potential role in leaf mineral homeostasis

**DOI:** 10.1093/jxb/erx067

**Published:** 2017-04-12

**Authors:** Noa Wigoda, Metsada Pasmanik-Chor, Tianyuan Yang, Ling Yu, Menachem Moshelion, Nava Moran

**Affiliations:** 1 The R.H. Smith Institute of Plant Sciences and Genetics in Agriculture, The R.H. Smith Faculty of Agriculture, Food and Environment, The Hebrew University of Jerusalem, Rehovot, Israel; 2 Bioinformatics unit, G.S.W. Faculty of Life Sciences, Tel-Aviv University, Israel; 3 College of Resources and Environmental Sciences, Nanjing Agricultural University, Nanjing, P.R. China; 4 State Key Laboratory of Tea Plant Biology and Utilization, Anhui Agricultural University, Hefei, P.R. China

**Keywords:** *Arabidopsis thaliana*, bundle sheath, C3 plant, membrane potential, membrane transport, mesophyll, patch clamp, ratiometric fluorescent probe, transcriptome, transporters

## Abstract

Under fluctuating ambient conditions, the ability of plants to maintain hydromineral homeostasis requires the tight control of long distance transport. This includes the control of radial transport within leaves, from veins to mesophyll. The bundle sheath is a structure that tightly wraps around leaf vasculature. It has been suggested to act as a selective barrier in the context of radial transport. This suggestion is based on recent physiological transport assays of bundle sheath cells (BSCs), as well as the anatomy of these cells.

We hypothesized that the unique transport functionality of BSCs is apparent in their transcriptome. To test this, we compared the transcriptomes of individually hand-picked protoplasts of GFP-labeled BSCs and non-labeled mesophyll cells (MCs) from the leaves of *Arabidopsis thaliana*. Of the 90 genes differentially expressed between BSCs and MCs, 45% are membrane related and 20% transport related, a prominent example being the proton pump AHA2. Electrophysiological assays showed that the major AKT2-like membrane K^+^ conductances of BSCs and MCs had different voltage dependency ranges. Taken together, these differences may cause simultaneous but oppositely directed transmembrane K^+^ fluxes in BSCs and MCs, in otherwise similar conditions.

## Teaser

The transcriptome analysis of leaf bundle sheath cells compared with mesophyll cells, supported by physiological assays, suggests a potential role of the bundle sheath in radial leaf transport.

## Introduction

The bundle sheath (BS) has been suggested as a critical control point for the radial transport of water and solutes between leaf vasculature and leaf mesophyll (reviewed by Sack and Holbrook, 2006; Leegood, 2008). Notably, in the leaf veins of a variety of plant species, there is evidence of a barrier to free movement of water and solutes out of the xylem and into the mesophyll apoplast, as seen by the accumulation of tracers of apoplastic flow in the xylem (Karley *et al.*, 2000). With regard to water transport, the Arabidopsis BS has been referred to as a bottleneck (Ache *et al.*, 2010) and its properties as a xylem-mesophyll barrier to water have been demonstrated (Pantin *et al.*, 2012; Shatil-Cohen and Moshelion, 2012; Sade *et al.*, 2014). An additional manifestation of the apoplastic barrier-to-water properties of the BS is the natural phenomenon of guttation, whereby drops of xylem sap appear on the tips or edges of leaves, usually at dawn. This demonstrates the ability of the BS to withstand the positive root pressure created in the xylem sap during the night, preventing water from flooding the air spaces within the mesophyll, while allowing its release via hydathodes at the leaf edges as guttation drops. As the radial passage of water through the BS is negligible, it stands to reason that this pathway is also a barrier to the passage of minerals. These minerals must therefore cross the membranes of BS cells (BSCs) on their way from the dead xylem conduits to the mesophyll symplast. Similarly, metabolites, ions and water flow through the BS during phloem loading in source leaves and phloem unloading in sink leaves (reviewed by Wigoda *et al.*, 2014).

Indeed, the selectivity of the transport mechanisms in the BSCs plasma membrane appears to be crucial in determining which solutes enter and move through the leaf symplast. For example, the BS of the C3 plant banana favored the transfer of K^+^ from the xylem to the mesophyll but rejected Na^+^ (Shapira *et al.*, 2009). The banana leaf vascular system was also able to withhold excess boron from the mesophyll during the day and to extrude it at night at the lamina edges, where the marginal vein lacks a complete BS (Shapira *et al.*, 2013). This eventually lead to tissue damage at the lamina edge. Similarly, in the C3 plant Arabidopsis, when plants were fed with elevated amounts of boron concentrations, only the leaf edges were initially damaged by the excessive accumulation of boron near the hydathodes at the vein endings, presenting the classical symptoms of boron toxicity (Lamdan *et al.*, 2012). This therefore demonstrated that boron was excluded from the mesophyll by BSCs. Conversely, in a small fraction of Arabidopsis plants in which the BS had faulty barrier functionality, as demonstrated by spontaneously flooded leaf mesophyll in the morning (Shatil-Cohen and Moshelion, 2012), boron toxicity caused necrosis inside the leaf prior to damage appearing at leaf edges. Boric acid is a small, membrane-permeable uncharged molecule that can easily cross an artificial membrane designed to desalinate seawater (Nadav, 1999). However its passage from leaf vasculature to the rest of the leaf appears to be restricted, at least to regions where the bundle sheath is intact.

In addition, BSCs appear to possess unique metabolic and transport properties (reviewed by Leegood, 2008). For example, in response to excess light, the BSCs of Arabidopsis leaves express ascorbate peroxidase (APX2) and accumulate hydrogen peroxide, while the neighboring mesophyll cells (MCs) produce singlet oxygen and superoxide (Fryer *et al.*, 2002). Arabidopsis BSCs also had a higher K^+^ concentration than MCs (Conn and Gilliham, 2010). These unique properties of BSCs imply a selective, epithelial-like barrier between BSCs and their neighboring MCs. In spite of the realization of the importance of this tissue, there are still few in-depth studies of BSCs.

The transcriptomes of only a few cell types in the Arabidopsis leaf have been characterized so far. For example, guard cell expression profiles were compared with those of MCs, resulting in the identification of 64 transcripts expressed preferentially in the guard cells. These comparisons also revealed abscisic acid (ABA) modulation, at the transcript level, of many known guard cell ABA signaling components (Leonhardt *et al.*, 2004). Another study of Arabidopsis cell-specific transcripts provided the localization of approximately 1000 expressed genes to either pavement, basal or trichome cells (Lieckfeldt *et al.*, 2008). An additional study compared the transcriptomes from four cell types: provascular/procambial cells from developing leaf veins, mature vascular cells, mesophyll cells and guard cells (Gandotra *et al.*, 2013). In yet another study Aubry *et al.* (2014) compared transcripts in Arabidopsis BSCs that were associated with ribosomes and were therefore in the process of translation. This BSC ‘translatome’ was compared with the whole leaf translatome.

The ‘resting’ transcriptome of Arabidopsis leaf BSCs, which also includes transcripts not associated with ribosomes, is still unknown. We hypothesized that the special role attributed to the BS as a selective barrier to transport, as well as other potentially unique functions of BSCs, are reflected in the BSC transcriptome. Our results confirmed this hypothesis following a comparison of the transcriptomes of individually hand-picked protoplasts of BSCs and MCs, which was further supported by physiological assays. These results emphasize the potential importance of BSCs in leaf transport regulation.

## Materials and methods

### Plant material

Transgenic *Arabidopsis thaliana* ecotype Landsberg (Ler) plants expressing green fluorescent protein (GFP) under the scarecrow (SCR) promoter (SCR:GFP) were grown as described by Shatil *et al*. (2011), with visual validation of GFP fluorescence performed on each individual SCR:GFP plant. Protoplast isolation was described previously (Shatil-Cohen *et al.*, 2011; Shatil-Cohen *et al.*, 2014). Briefly, protoplasts were isolated from pieces of Arabidopsis leaf, a few millimeters in size, with the abaxial epidermis peeled off and subject to a 20 min exposure to cell-wall-digesting enzymes. This was immediately followed with a wash to remove the enzymes and a brief gravimetric collection of the dispersed protoplasts, without centrifugation, to minimize tissue damage.

### Collection of single protoplasts for RNA extraction

**Fig. 1.**
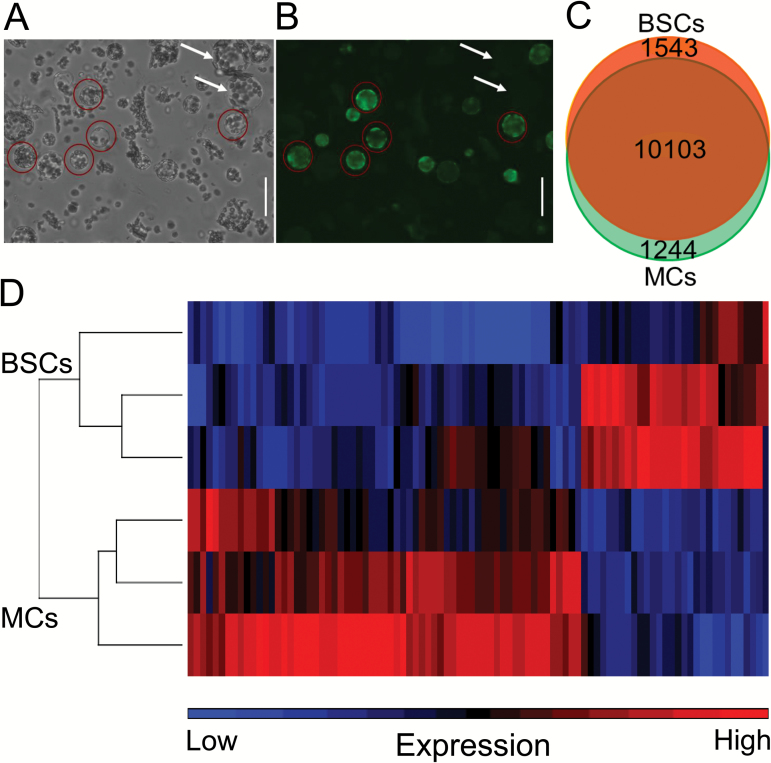
Genes expressed in BSCs and MCs. A. Isolated leaf protoplasts in white light. B. The same field of vision with the protoplasts excited by LED blue light (at 485 nm), showing protoplasts expressing GFP fluorescing green (at 520 nm). Red circles mark fluorescent BSCs. White arrows point to non-fluorescent MCs. Scale bar, 50 µm. (see Supplementary Protocols S1 for further details). C. An area-proportional Venn diagram of the genes expressed in BSCs and MCs at a level exceeding a threshold of log_2_(NREL)=2. Diagram generated using BioVenn. D. Heatmap of the 90 genes differentially expressed between BSCs and MCs, with expression levels of each gene (column) color-coded in each sample (row).

To limit variation in gene expression due to the circadian clock, all collections were performed between 10:00 AM to 1:30 PM. Single leaf protoplasts (Fig. 1) were collected manually with as little solution as possible using a homemade plastic suction pipette with a tip aperture of 200 µm (see Supplementary Protocols S1, Supplementary Fig. S1, Supplementary Movie S1 at *JXB* online), transferred into a 1.5 ml Eppendorf tube and flash-frozen in liquid nitrogen. The frozen samples were kept at -80ºC until further analysis.

### RNA extraction and amplification

#### RNA extraction

Total RNA was extracted from a pool of 20 single protoplasts of the same type using the ExpressArt LCM RNAready LCM RNA isolation kit (C#9001-A100, AmpTec, Germany) according to the manufacturer’s protocol, with the following exceptions: no vortex was used at any stage of the extraction to preserve RNA quality and a pooling procedure was included (Supplementary Protocols S2). RNA integrity was evaluated using an Agilent 2100 BioAnalyzer; the chip used was Eukaryote Total RNA Pico (Supplementary Fig. S2A). Only samples with good quality RNA were used.

#### RNA amplification and labeling

RNA was amplified three times. First, it was amplified twice using the ExpressArt mRNA amplification Pico kit (C#7399-A15, AmpTec, Germany). According to the manufacturer’s recommendation, the entire first amplification yield was amplified and only after the second amplification was the amplified RNA integrity further evaluated with the Agilent BioAnalyzer (Supplementary Fig. S2B). In the third round of amplification, RNA was labeled using the BioArray High Yield® RNA Transcript Labeling Kit (C#42655, Enzo Life Science, Inc.).

### Affymetrix array hybridization and evaluation

#### Array hybridization

Labeled RNA was hybridized to the Affymetrix GeneChip® Arabidopsis ATH1 Genome Array (ATH1-121501), at the Biological Services unit of the Weizmann Institute of Science, Israel. We used three biological replicates for each cell type, namely BSCs and MCs.

#### Array evaluation

Microarray analysis of Affymetrix CEL files was performed using Partek® Genomics Suite™, version 6.5 copyright 2010 (http://www.partek.com, last accessed 27 March 2017). Data were normalized and summarized with the robust multi-average method (Irizarry *et al.*, 2003). The gene expression data were then subject to ANOVA. Cluster analysis of the differentially expressed genes was obtained by Partek® Genomics Suite™ (using the cut-offs *P*<0.05 and absolute fold-change ≥1.5).

### Microarray gene expression data

The data discussed in this publication have been deposited in NCBI’s Gene Expression Omnibus (Edgar *et al.*, 2002) and are accessible through GEO Series accession number GSE85463 (https://www.ncbi.nlm.nih.gov/geo/query/acc.cgi?acc=GSE85463).

### Gene ontology analysis

Gene ontology (GO) analysis of the differentially expressed gene set was carred out in comparison with the entire Arabidopsis genome. This was performed using the Database for Annotation, Visualization, and Integrated Discovery (DAVID) online application (http://david.abcc.ncifcrf.gov, last accessed 27 March 2017) (Huang *et al.*, 2008; Huang *et al.*, 2009). Functional group categories with *P*<0.05 were considered signiﬁcantly enriched. Similar analysis was performed using other gene ontology tools, namely GOEAST (Zheng and Wang, 2008) and MapMan (http://mapman.gabipd.org/web/guest/mapman, last accessed 27 March 2017) (Thimm *et al.*, 2004).

 Inconsistencies regarding genes categorized as associated to GO:0022857 transmembrane transporter activity, according to TAIR or the DAVID application (Berardini *et al.*, 2004), were corrected manually. The added genes are marked within the relevant tables.

Venn diagrams were used to cross between the various gene sets (Venny, Oliveros JC, 2007; http://bioinfogp.cnb.csic.es/tools/venny/index.html, last accessed 27 March 2017). BioVenn was used to visualize area-proportional Venn diagrams (Hulsen *et al.*, 2008).

### Real-time PCR experiments

For quantitative real-time PCR (qRT-PCR), twice-amplified RNA (described in RNA extraction and amplification) from both cell types was reverse-transcribed into cDNA (iScript™ Advanced cDNA Synthesis Kit for qRT-PCR, Bio-Rad Laboratories, CA). Identical quantities of cDNA were used, as determined for each set of gene primers using a calibration curve. qRT-PCR was performed using a C1000 Thermal Cycler (Bio-Rad). Results were analyzed using Bio Rad CFX manager™ software. Dissociation curve analysis was performed at the end of each qRT-PCR reaction to validate the presence of a single reaction product and lack of primer dimerization. Expression levels of examined genes were normalized using two normalizing genes: *AT5G12240* and *AT2G07734*. Primers used for RT-PCR are detailed in Supplementary Protocols S3.

### Electrophysiological measurements

#### Protoplast selection

Individual BSCs protoplasts were selected for patch-clamp analysis based on their GFP fluorescence, with excitation at 490 nm and emission ≥535 nm, and their diameter being 25–30 µm. MCs were selected based on their lack of fluorescence and their diameter being 40–45 µm (as in Fig. 1 and in Shatil-Cohen *et al.*, 2011; Shatil-Cohen *et al.*, 2014). GFP-based selection was performed on a TMD-diaphot epifluorescence inverted microscope (Nikon, Japan) using light from a xenon lamp monochromator, Polychrome II (Till Photonics, Germany) included in the patch-clamp setup.

#### Pulse protocols and recording

Patch-clamp experiments were performed at a room temperature of 22–24 °C) using a Digidata 3122A interface, an Axopatch1C amplifier and pClamp 9 or 10 program suite, all from Axon Instruments (currently within Molecular Devices; https://www.moleculardevices.com/systems/axon-conventional-patch-clamp, last accessed 17 March 2017). This was used both for running the experiment and for analysis. Patch-clamp pipettes were pulled from borosilicate glass capillaries (Cat. #BF150-86-10, Sutter Instruments, Novato CA), ‘fire-polished’ against a heated filament, dipped in protamine sulfate (Sigma P-4380, 1% in water), dried and filled with an internal solution (see Solutions below). The pipette tip was then coated with wax. A drop with protoplasts was added to the bath solution (see Solutions below) in the bath. The protoplasts were allowed to settle for a few minutes. Whole-cell configuration was usually attained spontaneously without first obtaining a giga-seal. Holding the membrane at negative potentials frequently led to an increasing negative holding current and eventually to cell loss. Consequently a holding potential (E_H_) of 0 mV was selected. At E_H_ of 0 mV, the holding current usually decreased and stabilized within 10 min. Membrane currents were elicited from the protoplast membrane by repeated ‘sweeps,’ each containing 1 s long square voltage pulses decreasing from +120 mV to -160 mV at -20 mV steps. Each square pulse was followed by a 30 ms-long ramp, from +70 to -70 mV, referred to in the Results section as ‘G-V-testing ramp’. All of the aforementioned voltage values are nominal; the final analysis values were corrected for a liquid junction potential (LJP) of -23 mV (Supplementary Protocols S4, Fig. 4, Supplementary Fig. S3). The inter-sweep interval was 20–30 s. All experiments were performed in voltage-clamp mode. The capacitive transients were largely cancelled out using the compensatory circuit of the patch-clamp amplifier, with the series resistance (Rs) of the patch pipette compensated for at approximately 80%. The voltage error due to the residual Rs was less than 10% of the membrane potential. Currents were filtered at 500–1000 Hz and usually sampled at 2 kHz.

#### Analysis

All the membrane potential (E_M_) values mentioned in the results have been corrected for a LJP of -23 mV for the ‘low K^+^’ bath solution and -14 mV for the 30 mM K^+^ bath solution (Supplementary Protocols S4). The reversal potential (E_rev_) of the currents via channels activated by the square pulses was extracted from the intersection point of the currents elicited by the voltage ramps (arrowheads on the ramps in Supplementary Fig. S3). The membrane conductance (G) at the end of each pre-pulse, which therefore related to the E_M_ during that pulse, was obtained from the linear slope of the current-voltage (I-V) relationship during the subsequent brief G-V-testing ramp. G was divided by the protoplast’s surface area, yielding the specific conductance (G’) (Supplementary Protocols S4, Eq. S1). Individual G’-E_M_ relationships were fitted with the Boltzmann equation (Supplementary Protocols S4, Eq. S2; Hille, 2001). The individual best-fit values obtained from the fit were then averaged separately for each cell type. The fraction of open channels, P_O_, was calculated as in Supplementary Protocol S4, Eq. S3.

### Measurement of membrane fluorescence in individual protoplasts

For the ratiometric evaluation of protoplast membrane potential (Supplementary Protocols S5), we used the dual-excitation fluorescent dye di-8-ANEPPS (Pucihar *et al.*, 2009). The dye and pluronic acid, at final concentrations of 30 µM and 0.05% (w/v), respectively, were added to protoplasts bathing in a ‘low K^+^’ solution (see Solutions below). Here the protoplasts were incubated at 4°C for 12 min and then washed three times with the same solution without both the dye and pluronic acid. In some experiments, a ‘vanadate-10-K^+^ solution’ (see Solutions below), was used for washing and during the image acquisition. Images were acquired using an inverted epifluorescence microscope (Olympus-IX8 Cell-R; Olympus, Japan; http://www.olympus-global.com, last accessed 27 March 2017) equipped with a 40x objective with a numerical aperture of 1.15, a mercury lamp as an excitation light source and a CCD 12-bit Orca-AG camera (Hamamatsu, Japan; http://www.hamamatsu.com). One transmitted light image and two fluorescence images were acquired sequentially at two excitation wavelengths of 438 nm and 531 nm and at a single emission wavelength of 593 nm (Supplementary Fig. S4, Supplementary Protocols S5). The images were processed to yield a mean ratio (Supplementary Protocols S5).

### Solutions for protoplast isolation, electrophysiology and imaging

In all solutions listed below, pH was adjusted with an N-methyl D-glucamine base and osmolality was adjusted with D-sorbitol.

Wash solution: 1 mM CaCl_2_, 10 mM KCl, 6 mM MES, at pH 5.56 and osmolality 600 mOsm.

Enzyme solution: 0.8% (w/v) cellulose onozuka R-10 (Y), 0.1% pectolyase (Sigma), 0.5% bovine serum albumine. A powder of 0.5% polyvinylpyrrolidone was added to the wash solution immediately before use.

Bath ‘low K^+^ solution’: 5 mM KCl, 1 mM CaCl_2_, 4 mM MgCl_2_, 10 mM MES, at pH 5.6 and osmolality 435 mOsm.

Bath ‘30-K^+^ solution’: 25 mM K-gluconate, 5 mM KCl, 1 mM CaCl_2_, 4 mM MgCl_2_, 10 mM MES, at pH 5.6 and osmolality 435 mOsm.

Pipette ‘internal’ solution: 112 mM K-gluconate, 28 mM KCl, 4 mM MgCl_2_, 10 mM HEPES, at pH 7.5 and osmolality 480 mOsm. Just before the experiment, final concentrations of 2 mM K_2_-ATP and 2 mM K_4_-BAPTA were added to the pipette solution, resulting in a free Ca^2+^ concentration of <2 nM. The solution was filtered through a sterile 0.22 µm PVDF filter (Millipore, Ireland, SLGVO33RS).

K_2_ATP (Adenosine 5’-triphosphate; Sigma cat. #A-8937): a 200 mM stock solution was prepared using deionized distilled water, aliquoted and kept frozen at -20 ^o^C for up to a month.

K_4_BAPTA (K_4_-1,2-bis (o-aminophenoxy) ethane-N;N;N;N-tetraacetic acid; Sigma cat. #B-1204): a 200 mM stock solution was prepared using deionized distilled water, aliquoted and kept frozen at 20 ^o^C for up to a month.

‘Vanadate-10-K^+^ solution’: 1 mM Na_3_VO_4,_ 5 mM KCl, 5 mM KNO_3_, 1 mM CaCl_2_, 4 mM MgCl_2_, 10 mM MES, at pH 5.6 and osmolality 435 mOsm.

Vanadate (sodium orthovanadate, Na_3_VO_4_, BHD Chemicals Ltd. cat. #30194): the solution was depolymerized by boiling (https://www.wikiformulation.org/protocols/preparation-of-sodium-orthovanadate-solution/10011, last accessed 27 March 2017), aliquoted and kept frozen until use.

Di-8-ANEPPS (Enzo Life Sciences, Inc. cat. #ENZ-52204, Farmingdale, New York, USA): a 10 mM stock solution was prepared by adding 843 μL of DMSO to 5 mg of the dye. Aliquots of the stock solution were stored at -20 °C.

Pluronic acid (Sigma-Aldrich, cat. #F127): a 20% stock solution in DMSO (w/v) was kept in aliquots at -20 °C.

### Statistics

Means are presented with their standard errors unless indicated otherwise, with *n* indicating the number of cells averaged. Differences between means were deemed significant if, using a two-sided Students *t*-test, *P*<0.05.

## Results

### Isolation of ultra-pure MC and BSC protoplasts for transcription analysis

An essential requirement for obtaining an accurate transcriptional profile is the purity of the tissue or cellular source for RNA extraction. We therefore opted for a manual collection of individual protoplasts of non-fluorescing MCs and GFP-fluorescing BSCs with visual, cell-by-cell, validation of the collection purity. Subsequently, we extracted RNA from three batches of 20 protoplasts of each cell type (see Materials and methods). We used the Affymetrix ATH1 GeneChip, which contains probes for approximately 22 800 genes of Arabidopsis, to investigate the gene expression patterns of BSC genes compared to MCs. One RNA extract was used per microarray i.e. there were three biological repeats per a cell type.

### Transcriptome of BSCs and MCs

In total, we identified 12 890 expressed genes, of which 78% are common to the MC and BSC transcriptomes (Fig. 1C), with log_2_(normalized raw expression level), i.e. log_2_(NREL), ≥2. This threshold is well above background and was the median expression level in both cell types i.e. half of the genes had a log_2_(NREL) below two, and half above two. To compare the expression levels in the BSCs and MCs, we counted the expressed genes and grouped them according to their log_2_(NREL) (see explanation in Supplementary Fig. S5 legend). The number of genes at each level-of-expression group i.e., with log_2_(NREL) between one and two, between two and three, and so on, was roughly similar in both cell types (Supplementary Fig. S5). We determined the non-stringent but still biologically meaningful cut-off threshold of at least 50% change in transcript level, with an absolute fold-change≥1.5 and *P* <0.05. Based on these cut-off values and using the Partek® Genomics Suite™, 90 genes showed differential expression between BSCs and MCs (Supplementary Data S1). About a third of these genes were expressed at a higher level in BSCs compared with MCs, while two thirds had a lower expression level in BSCs compared with MCs (Fig. 1D).

### Functional differentiation between BSCs and MCs

**Fig. 2.**
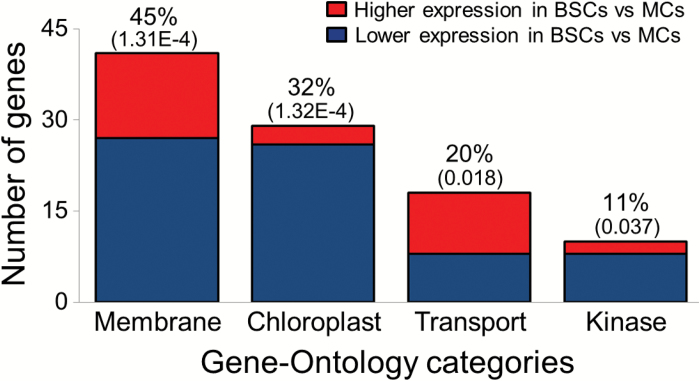
Enriched gene ontology (GO) functional categories of genes differentially expressed between BSCs and MCs. Columns: the number of differentially expressed genes in each enriched functional category. Above each column, the numbers of these genes out of the total pool of 90 differentially expressed genes are shown as percentages. The *P*-value for each enriched GO category is also shown in parentheses above each column. Colors show the proportions of genes with higher or lower expression in BSCs versus MCs in each category. Note that a gene may appear in more than one functional category.

We performed gene ontology (GO) analysis for functional enrichment on the above 90 genes (Fig. 2). This was performed using the DAVID online application (Huang *et al.*, 2008, 2009). Of the genes that showed differential expression between BSCs and MCs, 45% were related to the membrane (GO:0016020) and 32% were associated with the chloroplast (GO:0009507). Most of the genes associated with chloroplast functionality were expressed at lower levels in BSCs compared with MCs (Fig. 2), which is in accordance with the lower numbers of chloroplasts found in BSCs when compared with MCs in Arabidopsis (Kinsman and Pyke, 1998; Shatil-Cohen *et al.*, 2011). Interestingly, a few genes associated with chloroplast functionality had a higher expression in BSCs when compared with MCs (Fig. 2). These genes were: (a) *DIN10* (*AT5G20250*), which showed a fold change of 5.63, with *P*=0.035. It encodes a member of the glycosyl hydrolase family 36 and is located in the chloroplast; (b) *CRR2* (*AT3G46790*), which showed a fold change of 2.34, with *P*=0.032. It encodes a protein involved in the maturation steps i.e. inter-genic processing, of chloroplast RNA and is essential for the translation of NADH dehydrogenase ND2 (Hashimoto *et al.*, 2003); (c) two non-annotated genes identified by the same probe, *AT4G31030*, which encodes a putative mitochondrial membrane lipoprotein and *AT4G31040*, which encodes a CemA-like proton extrusion protein-like protein. Together these two genes showed a fold change of 4.54, with *P* =0.031.

A fifth of the genes that showed differential expression between BSCs and MCs were associated with transport (GO:0006810; Fig. 2). Of these, two-thirds were associated with transporter activity (GO:0005215; Supplementary Table S1).

Ten genes, making up 11% of the differentially expressed genes, had functionality associated with protein kinase activity (GO:0004672; Fig. 2). Of these 11 genes, eight genes had lower expression levels in BSCs compared with MCs. Only two genes in this group were expressed at a higher level in BSCs: *PERK8* (*AT5G38560*), which encodes a proline-rich extensin-like receptor kinase and showed a fold change of 1.58, with *P*=0.008, and a non-annotated gene (*AT3G28040*), which encodes a leucine-rich receptor-like protein kinase and showed a fold change of 1.63, with *P* =0.045.

Functional enrichment was re-analyzed using other gene ontology tools, such as GOEAST and MapMan (Supplementary Fig. S6). The results of these analyses are consistent with those of the analysis performed using the DAVID online application.

### Transporter gene classification by expression level

**Fig. 3.**
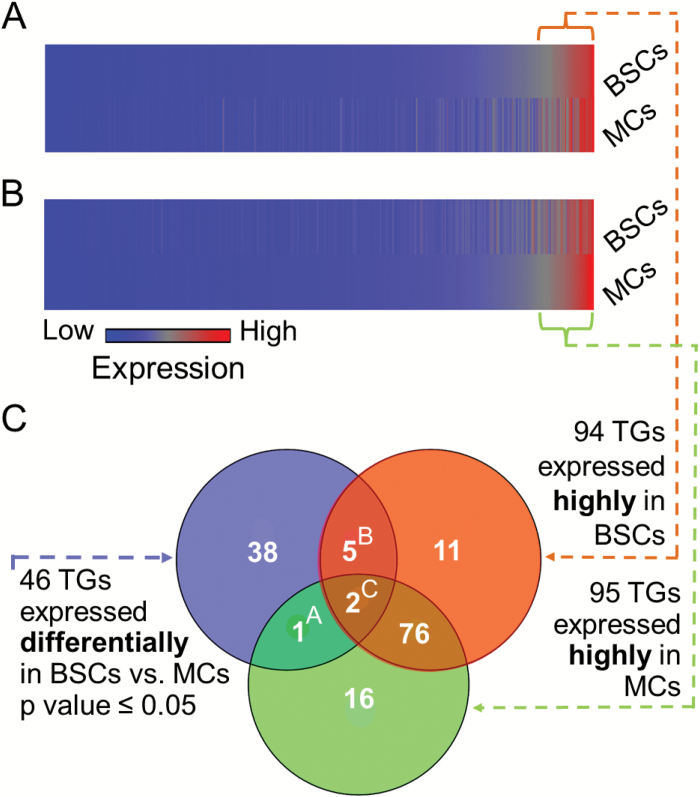
Identifying transporter genes with high expression levels. Color-coded representation of mean log_2_(NREL) of 862 transporter genes (TGs) associated with transmembrane transporter activity (GO:0022857). A column across the whole bar represents a single TG in both cell types. A. The 862 TGs sorted in BSCs according to descending expression levels. The bracket marks the highly expressed TGs with a log_2_(NREL)≥3.5. B. The same as in A but for TGs sorted in MCs. C. Venn diagram of TGs differentially expressed between BSCs and MCs and of the highly expressed TGs in BSCs and MCs. Numbers in circles are the number of genes in each set. Superscripts refer to subsets of TGs listed in Supplementary Table S2. The dashed arrows are to aid visualization.

We analyzed all the genes that are functionally associated with transmembrane transporter activity (GO:0022857, according to TAIR) and examined their averaged log_2_(NREL) in the three replicates (microarrays) for each cell type (Fig. 3A, B). We recognized 862 genes related to transmembrane transporter activity (GO:0022857); henceforth referred to astransporter genes, TGs. These genes comprise 95% of the 906 genes of this functionality that could have potentially been recognized by the Affymetrix ATH chip (which is less than the 1018 known genes related to transmembrane transporter activity, GO:0022857, in the TAIR Gene Ontology database).

We sorted the 862 TGs according to their mean log_2_(NREL), in BSCs (Fig. 3A, top half-bar) and in MCs (Fig. 3B, bottom half-bar). Note that both cell types exhibit a similar distribution pattern when sorted according to gene expression level. Thus, in both MCs and BSCs, about 10% of the 862 recognized TGs were highly expressed, with log_2_(NREL)≥3.5, while about 90% of the recognized TGs had low expression levels, with log_2_(NREL)≤3.5 (Fig. 3A, B). Importantly, 30% of the most highly expressed transporter genes, 33 out of 111 genes, are different between the BSCs and MCs (Fig. 3C).

### Transporter genes expressed differentially between BSCs and MCs

We re-analyzed those of the 862 TGs, which were expressed differentially between BSCs and MCs, with a cut-off of *P*≤0.05 but without a restriction on fold change. This resulted in 46 genes (Fig. 3C). Of these 46 TGs, 19 TGs had lower expression levels in BSCs compared with MCs, while 27 had higher expression levels in BSCs compared with MCs (Supplementary Table S2). Approximately 89% of the genes with the higher expression levels in BSCs were related to the membrane and five of these genes were directly related to proton transport, namely *AHA2* (*AT4G30190*), *ZIFL1 (AT5G13750*), *ATSUC8* (*AT2G14670*), *CHX24* (*AT5G37060*), and *CHX14* (*AT1G06970*).

To examine the expression levels of these 46 transporter genes, we crossed this set with the sets of transporter genes that showed high expression levels i.e. mean log_2_(NREL)≥3.5), in each cell type (Fig. 3A, B). Among the TGs that showed differential expression between BSCs and MCs, we identified three groups: group A (Fig. 3C, Supplementary Table S2) contained only the voltage-gated anion channel gene *VDAC3*, which was expressed at a high level only in MCs; group B (Fig. 3C, Supplementary Table S2) contained five genes, which were expressed at a high level only in BSCs. This group included *PDE318*, which encodes a ferrous iron transmembrane transporter; *RAN1*, which encodes a copper-transporting ATPase; and three non-annotated genes, *AT5G17850*, which encodes a sodium/calcium exchanger; *AT5G19500*, which encodes a putative chloroplast-localized amino acid transporter; and *AT5G05310*, which encodes an ATP/ADP translocase; group C (Fig. 3C, Supplementary Table S2) contained two genes, which were expressed at a high level in both BSCs and MCs. This group included *ATTIM17-1*, which encodes a subunit of a translocase complex of the mitochondrial inner membrane and *AHA2*, which encodes a proton-exporting ATPase of the plasma membrane.

 The relative levels of *AHA2* in the BSCs and MCs and in the microarray analysis were closely replicated by qRT-PCR (Supplementary Fig. S7), which was performed on twice-amplified RNA from the BSCs and MCs. The differential expression of additional representative genes was also verified by qRT-PCR; namely *PSBQA* that shows elevated expression in MCs, *RAN1* that shows elevated expression in BSCs and *AT2G32170* that shows similar expression in both cell types.

### Physiological differences between BSCs and MCs

#### A. Comparison of ion channel activity in BSCs and MCs from patch-clamp assays

Based on the observed difference in K^+^ accumulation in BSCs and MCs (Conn and Gilliham, 2010), we expected a difference in K^+^ transporters, possibly even in K^+^ channel transcript levels. We found a significant but small difference of less than 1.5-fold in the expression of two K^+^ carriers from the AtT2/KUP family (Supplementary Table S2). These K^+^ carriers were more prevalent in the BSCs: *AtKT2* (*AT2G40540*) was 1.11-fold higher in the BSCs than in the MCs and *KUP11* (*AT2G35060*) was 1.10-fold higher in BSCs than in MCs (Supplementary Table S2).

Using the threshold of a 1.5-fold difference, we found no difference in transcript levels of K^+^ transporters, including K^+^ channels (Supplementary Table S3). However there was a difference in the transcripts of kinases (Fig. 2).

With regard to K^+^ channels, we wondered whether post-translational channel modulation, rather than transcript levels, distinguishes between the cell types. For example, channel activity may be modulated differently in the two tissues by kinases and/or by AT1G49740, a PLC-like phosphodiesterase with phospholipase C activity. The expression level of AT1G49740 was 67% greater in BSCs than in MCs, with a fold change of 1.67 and *P*=0.036. AT1G49740 could therefore cause a difference in the lipid composition of the membranes of BSCs and MCs, possibly leading to differences in channel activity (see for example Liu *et al.*, 2005; Ma *et al.*, 2009; Wigoda *et al.*, 2010). We therefore compared ion channel activity in protoplasts similar to those used for the transcript analyses. We focus here on time-dependent currents activated by hyperpolarizing potentials and deactivated by depolarizing potentials.

### BSC and MC membrane currents

The mean diameter of the isolated BSC protoplasts selected for these experiments was 27.4 ± 1.2 µm ±SD (*n*=12), which is similar in size to that used by Shatil-Cohen *et al.* (2011). The mean diameter of the isolated MCs protoplasts was 43.5 ± 2.4 µm ±SD (*n*=6). We studied the ion fluxes in membranes of isolated protoplasts using patch-clamp in the whole-cell configuration. In the exploratory phase where the holding current was close to null and stable, we set the E_H_ at -23 mV, which was already corrected for the LJP (see Materials and methods; Supplementary Fig. S3A). These conditions were optimal for most cells and were therefore used as routine to ensure consistency. We applied voltage pulses and measured the current carried by ions moving through channels in the plasma membrane. In some cells, voltage pulses to potentials more positive than the E_H_ evoked time-dependent outward (positive) currents that decayed, i.e. deactivated, during the constant voltage. Voltage pulses to potentials more negative than the E_H_ evoked inward (negative) increasing time-dependent currents. This was true of protoplasts from both cell types (Supplementary Fig. S3B).

### Channel selectivity

The current linearity during the fast G-V-testing voltage ramp demonstrated that its brevity was sufficient to avoid any change in the number of open channels during the ramp (Supplementary Fig. S3C). Thus, the different slopes of the current ramps during the same applied voltage ramp were due to the different fractions of these channels opened by the pre-pulses (Supplementary Fig. S3B). The E_M_ at the crossover point of the current-ramps (Supplementary Fig. S3C, arrowheads), at which the currents were equal irrespective of the number of the pre-opened channels, was the reversal potential (E_rev_) of the current through these open channels.

The mean E_rev_ was -31 ± 7 mV ±SD (*n*=7), which was considerably more depolarized than the calculated K^+^ equilibrium (Nernst) potential (E_K_) of -81 mV in these experimental solutions. All other ions in the experimental solutions had calculated Nernst potentials ≥0: the calculated chloride ion equilibrium potential (E_Cl_) was +21 mV, the calculated calcium ion equilibrium potential (E_Ca_) was +176 mV, and the calculated magnesium ion equilibrium potential (E_Mg_) was 0 mV. In light of this, we can conclude that the membrane permeability when the channels were open was dominated by permeability to K^+^.

Elevating K^+^ concentration in the bath from 5 mM to 30 mM using the ‘30-K^+^ solution’ (see Materials and methods), shifted the calculated E_K_ to -38 mV, while the calculated Nernst potentials for all other permeant ions remained unchanged. The mean E_rev_ shifted by 14 mV to -17 ± 2 mV ±SD (*n*=9). Although the E_rev_ was still more depolarized than the E_K_, both the shift (*P*<0.001) and the fact that the E_rev_ was negative (*P*<0.01) indicated that during the hyperpolarizing pulses the membrane permeability was dominated by permeability to K^+^.

### Channel maximum conductance and gating properties

**Fig. 4.**
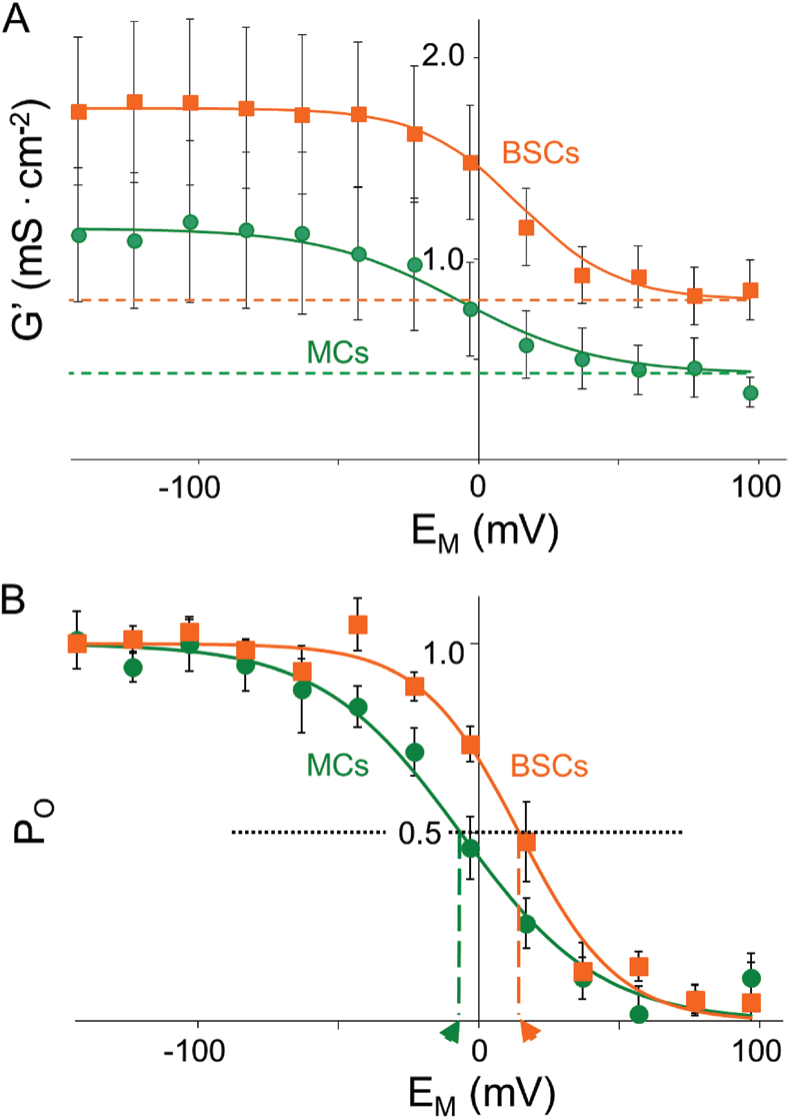
Channel gating properties in BSCs and MCs. A. Steady-state conductance-voltage (G’-E_M_) relationships. Squares and circles denote the mean ±SE specific membrane conductance (G’) for BSCs (*n*=8) and MCs (*n*=4), respectively. These were calculated from at least four independent experiments using Eq. S1 (see Supplementary Protocols S4). The solid line values were calculated using Eq. S2 (see Supplementary Protocols S4) using the mean Boltzmann best-fit parameters for BSCs or MCs. The horizontal dashed lines indicate the fitted basal G’ values. B. Voltage dependence of the fraction of open channels i.e. P_O_-E_M_ relationships. Squares and circles denote mean ±SE P_O_ for the cells in A. The solid line values were calculated using Eq. S3 (see Supplementary Protocols S4) using the mean best-fit z and E_1/2_ for BSCs or MCs, as in A. Horizontal dotted line denotes P_O_=0.5. Vertical dashed lines and arrowheads denote E_M_=E_1/2_.

The steady-state voltage dependence of the specific membrane conductance, the G’-E_M_ relationship (Fig. 4A), was obtained by plotting the slopes of the current-voltage relationship during the brief G-V-testing ramp (Supplementary Fig. S3C), normalized to the protoplast surface area (Supplementary Protocols S4, Eq. S1), against the E_M_ during the preceding pre-pulse.

The individual protoplast sigmoidal G’-E_M_ relationships were fitted with the Boltzmann equation (Fig. 4A; Supplementary Protocols S4, Eq. S2). This yielded the asymptotic, voltage-invariant best-fit basal conductance (G’_b_) and the maximum attainable conductance (G’_max_) (Supplementary Protocols S4), which were for BSCs 0.8 ± 0.14 and 1.75 ± 0.39 mS^.^cm^-2^ ±SE (*n*=8), respectively, and for MCs 0.43 ± 0.15 and 1.15 ± 0.35 mS^.^cm^-2^ ±SE (*n*=4), respectively. The mean of differences between the individual G’_b_ and G’_max_ (G’_max_net_) was 0.95 ± 0.33 and 0.72 ± 0.25 mS^.^cm^-2^ for BSCs and MCs, respectively.

The other two best-fit parameters, E_1/2_ and z, reflect the voltage dependence of G’ i.e. of channel gating (Supplementary Protocols S5, Eq. S2), and were 14.5 ± 4.5 mV and 1.46 ± 0.18 for BSCs, respectively, and -6.5 ± 3.5 mV and 1.03 ± 0.4 for MCs, respectively.

Thus, while G’_max_net_, G’_b_, and z did not differ between the two cell types, E_1/2_ was 21 mV more positive in BSCs compared with MCs (*P*<0.001). This 21 mV difference in E_1/2_ means that for a given membrane potential, except at the plateau, a larger fraction of the channels in BSCs are open when compared with the fraction of channels open in MCs (Fig. 4B).

#### B. Comparison of membrane potentials using ratiometric fluorescence imaging

Our transcriptome data revealed over a three-fold higher transcript level of AHA2 in BSCs compared with MCs. We therefore hypothesized that BSCs would also have higher hydrogen ion-pumping activity across the plasmalemma and consequently a more hyperpolarized membrane potential, especially when bathed in the same medium. To test this hypothesis, we assayed the membrane potential ratiometrically, using the fluorescent potentiometric dye di-8-ANEPPS (see Materials and methods).

**Fig. 5.**
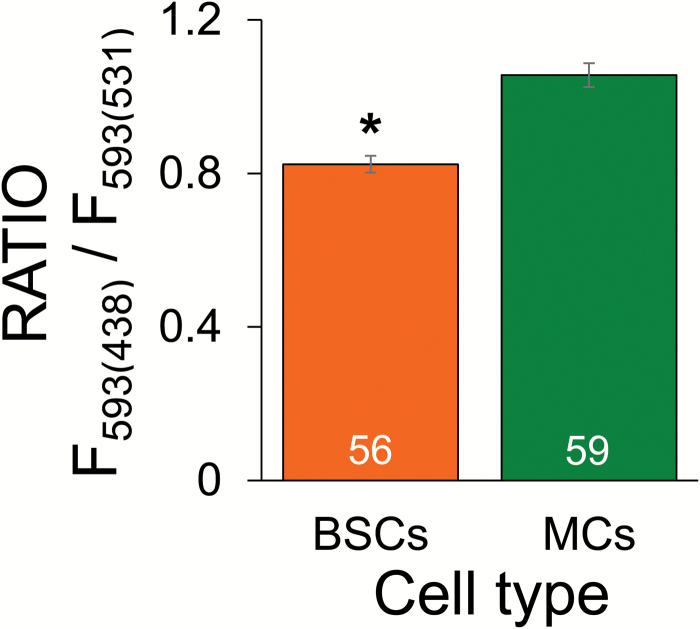
Membrane fluorescence ratio determined for BSC and MC protoplasts stained with the potentiometric probe di-8-ANEPPS. The mean ±SE fluorescence ratio values from the indicated number of protoplasts of each cell type in six independent experiments. Imaging was carried out by sequential excitation at 438 nm and 531 nm with emission at 593 nm. Note the smaller ratio for the BSCs, *P*<0.001.

As we expected, the fluorescence imaging of stained protoplasts yielded different results for the two cell types: the calculated ratio values were higher for the MCs than for the BSCs (Fig. 5). Lower ratio values correspond to lower i.e. more hyperpolarized transmembrane potentials (Zhang *et al.*, 1998; Breygina *et al.*, 2009; Pucihar *et al.*, 2009; Bachtel *et al.*, 2011). The lower ratio values in BSCs was therefore consistent with our hypothesis that BSCs have a more hyperpolarized E_M_ than MCs.

We performed additional experiments to rule out the possibility that the different ratio values are due to other cell-type dependent properties, such as membrane composition (Starke-Peterkovic *et al.*, 2006). We assayed the cells in the presence of 1 mM orthovanadate, assuming that blocking the proton pumps would depolarize both cell types and abolish the membrane potential difference between them. To enhance this depolarization, we added 5 mM KNO_3_ along with vanadate, to aid in dissipating the membrane potential and depolarizing the cells via proton-coupled K^+^ and nitrate ion transport. Additionally, nitrate ions may depolarize the cells by activating anion efflux, as in root parenchyma cells (Kohler *et al*., 2002). As predicted, the ratio values of BSCs increased to a value similar to that of MCs (Supplementary Fig. S8), supporting the notion that di-8-ANEPPS reported a true membrane potential difference between the cells when assayed without the depolarizing agents.

## Discussion

In this study, we highlighted the unique transporter-related properties of BSCs by comparing them with MCs. Physiological processes are often restricted to specific tissue types or even to individual cell types. To enhance the understanding of biological processes associated with a specific cell type, such as BSCs, single-cell techniques are required. Cell-specific studies can detect many low-abundance and cell-specific transcripts, which are undetectable in whole tissue or whole organ samples (Bailey-Serres, 2013). This observation emerged from comparisons of the rice BSC transcriptome to the whole-leaf transcriptome in the rice genome atlas (Jiao *et al.*, 2009). We used protoplasts to identify cell type-specific expression patterns. In order to reduce any impact on the results from protoplast preparation, we used an improved and very short extraction protocol of ~25 min with minimum tissue injury and without centrifugation (Shatil-Cohen *et al.*, 2014). Moreover, both cell types were extracted together. This identical treatment of all tissue samples enables data to be relatively compared between the tissues, for examples in terms of fold-changes in gene expression (Spencer *et al.*, 2007).

In general, the number of differential genes identified depends on the tissues ans conditions being compared. BSCs and MCs are both green cells in the leaf and at the protoplast level are barely distinguishable unless labeled, for example with specifically targeted GFP. This similarity may be one of the reasons behind detecting only 90 differentially expressed genes. Notably, even smaller numbers have been reported from microarray experiments. For example, in Arabidopsis seedlings Mustroph *et al.* (2009) identified only 27 enriched mRNAs in the root cortex meristem and 20 enriched mRNAs in the trichome-targeted (pGL2) population compared with all other non-overlapping cell types in the same organ.

Another reason for the low number of differential genes could be variability in our microarray results (Fig. 1D). This was likely related to the large biological variability among the relatively small samples i.e. 20 individual cells per sample, as well as the unavoidable three rounds of RNA amplification. We also cannot exclude the introduction of some variability by the potentially different responses of the two cell types to their enzymatic treatments during isolation, despite use of the same brief and gentle procedure applied simultaneously to all the cells. In addition, microarray technology is known to detect low copy mRNAs with a lower sensitivity than next generation sequencing technology. It is thus possible that genes with low levels of expression could have escaped detection in our work.

Notwithstanding the low number of the differentially expressed genes that we detected, some of our results do support the work of others. Similar to our results, Aubry *et al.* (2014) also found a poor representation of photosynthesis-related genes and an over-representation of transport categories in BSCs. Other groups who explored the BSC transcriptome of maize also noted enrichment in transport-related genes (Li *et al.*, 2010; Chang *et al.*, 2012; Tausta *et al.*, 2014).

Our finding that 45% of the genes that were differentially expressed between BSCs and MCs are membrane related and 20% are transport related, suggests that BSCs are points of control for the radial transport of solutes. Furthermore, our finding that 30% of the most highly expressed transporter genes are different between the BSCs and MCs (Figs. 3A, 3B) strengthens the notion that BSCs and MCs very likely play distinctly different roles in the transfer of solutes and water.

Our transcriptome comparison between BSCs and MCs showed no difference in transcript levels of K^+^ channels (Supplementary Table S3). This prompted electrophysiological assays seeking a possible difference between the two cell types at the functional level of their channels. Voltage-gated K^+^ channels are essential for the distribution of K^+^ in the entire plant (Dreyer and Uozumi, 2011). Unsurprisingly, we identified the hyperpolarization-evoked currents as K^+^ currents, affirming that K^+^ channels in BSCs and MCs were the most dominant contributor to membrane permeability. However although the currents reversed at a negative membrane potential (E_rev_) and only K^+^ had a negative calculated Nernst potential (E_K_), the E_rev_ was still more positive than the E_K_. This result resembled positive E_rev_ deviations from E_K_ in whole-cell inward K^+^ currents recorded in *Brassica chinensis* pollen protoplasts (Fan *et al.*, 1999). These deviations indicate a contribution of at least one other ion type to the recorded currents. We recorded current in a whole-cell configuration and hence through an ensemble of channels in the cell membrane. Consequently, we could not tell whether the additional ion types contributed to the current via the same or different channel types. This remains to be resolved.

The BSCs and the MCs differed in the voltage-dependence of their inward rectifier channels, as seen in their P_o_-E_M_ relationships. The rightward shift of the BSC P_o_-E_M_ curve relative to the MC P_o_-E_M_ curve indicates that a larger fraction of these channels were open in BSCs at a comparable range of activation. For example, at -23 mV, the value of E_H_ in our experiments, 89% of the channels in BSCs were open compared with 66% of the channels in MCs. Notably, the mean E_1/2_ of both cell types, 14.5 and -6.5 mV for BSCs and MCs, respectively (Fig. 4B) was much more positive than the presumed prevailing negative membrane potential inside plant cells (-120 to -160 mV), (Sze *et al.*, 1999). This may have resulted from a superposition of currents from the variously phosphorylated AKT2 channels (Michard *et al.*, 2005*a*, *b*; Sandmann *et al.*, 2011), with or without currents from KAT1 and/or KAT2 and/or AKT1 channels. These four K^+^ channel genes were expressed in both cell types (Supplementary Table S3). In addition, the larger rightward shift of E_1/2_ in BSCs could correspond to a larger fraction of AKT2 being phosphorylated or otherwise post-translationally modified.

**Fig. 6.**
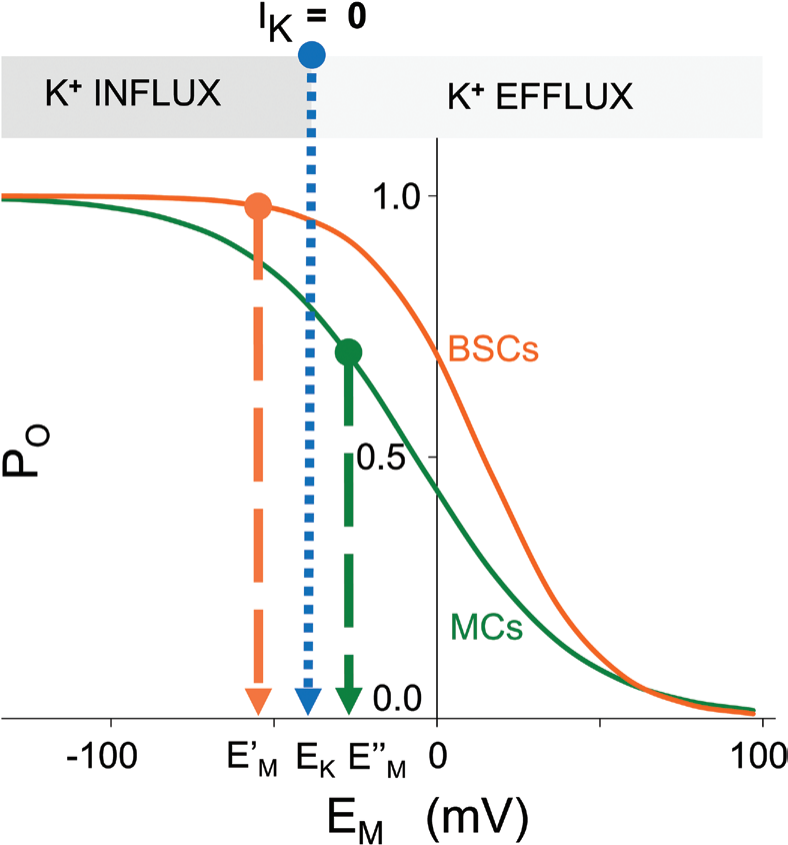
Hypothesized directions of K^+^ flow across membranes of BSCs and MCs. Voltage dependence of the fraction of open channels i.e. P_O_-E_M_ relationships, of BSCs and MCs from Fig. 4B. The different E_1/2_, E’_M_ and E’’_M_ values denote the different membrane potentials of the two cell types. E’_M_<E’’_M_ based on Fig. 4. E_K_ is the E_M_ with zero net K^+^ flow. The placement of the E’_M_ and E’’_M_ relative to E_K_ is an example indicative of what could occur in conditions resembling our experiment.

We can envision that this difference in the voltage range of channel activation has functional implications and therefore physiological relevance, especially when one considers that BSCs had a more negative E_M_ than MCs (Fig. 5). We attribute the more negative E_M_ to the larger hyperpolarizing activity of the proton pump in BSCs, based on the 3-fold higher expression of AHA2 in BSCs, relative to MCs. All other conditions being equal, including G_max_net_, a larger K^+^ flux would be expected in BSCs at a P_O_ transition range due to the larger fraction of these channels open in BSCs compared with MCs. Moreover, with the more negative E_M_ in BSCs (E’_M_) and the less negative E_M_ in the MCs (E”_M_), the electrochemical potential driving K^+^ fluxes could be different in both cells. This could cause, for example, K^+^ influx in BSCs and K^+^ efflux in MCs (as suggested in Fig. 6).

A sufficiently hyperpolarized plasma membrane, due to the activity of the plasma membrane H^+^-ATPase, could enable inward K^+^ channel-mediated flux of K^+^ even with an external K^+^ concentration as low as 10 µM (Britto and Kronzucker, 2008). Such a scenario could perhaps explain the larger accumulation of K^+^ in the BSCs (as indicated by Conn and Gilliham, 2010). The larger hyperpolarization in the BSCs when compared with the MCs could also contribute to larger electrochemical potentials driving cation influx through other channel proteins. Moreover, and no less importantly, the larger potential energy that the H^+^-ATPase most likely generates across BSC membranes as a proton electrochemical potential gradient (Sze *et al.*, 1999; Palmgren, 2001; Haruta and Sussman, 2012) is capable of more effectively driving a large number of transport processes by secondary-active transporters. Candidates for such proton motive force (PMF)-powered carriers could be the two transporters from the 13-gene family of K^+^ uptake permeases, AtKT2 and AtKUP11, found to be expressed about 10% more in BSCs (namely, below our defined 1.5-fold threshold; Supplementary Table S2). The functionality of these K^+^ carriers has been demonstrated by expression in *Saccharomyces cerevisiae* (AtKT2, Quintero and Blatt, 1997) or in *E. coli* (KUP11, Ahn *et al.*, 2004; and AtKT2, Elumalai *et al.*, 2002) and their homolog HAK1 has been shown to function as a K^+^/H^+^ symporter in *Neurospora crassa* (Haro *et al.*, 1999). Possibly, even the slight but significantly higher expression levels of AtKT2 and AtKUP11 in BSCs could possibly be an underlying factor in the above mentioned higher accumulation of K^+^ in BSCs compared with MCs (Conn and Gilliham, 2010). Thus, even if transporters or channels do not differ much, or at all, between BSCs and MCs, the difference in AHA2 may result in pronounced differences between the two cell types in the transport and efficient absorption of many nutrients and metabolites. Such physiological differences remain to be determined.

## Supplementary Data

Supplementary data are available at *JXB* online.

Fig. S1. Manual collection of individual protoplasts for expression profiling.

Fig. S2. BioAnalyzer RNA integrity test.

Fig. S3. Time- and voltage-dependent whole-cell currents in a BSC and an MC.

Fig. S4. Arabidopsis leaf protoplasts stained with the potentiometric probe di-8-ANEPPS.

Fig. S5. Numbers of genes expressed in BSCs and MCs grouped according to mean log_2_(NREL).

Fig. S6. GOEAST and MapMan ontology analysis of genes differentially expressed between BSCs and MCs.

Fig. S7. qRT-PCR validation of gene expression in BSCs and MCs.

Fig. S8. The effect of depolarizing agents on the fluorescence of BSC and MC protoplasts stained with the potentiometric probe di-8-ANEPPS.

Table S1. Genes associated with transporter activity (GO:0005215) that are differentially expressed between BSCs and MCs.

Table S2. Genes associated with transmembrane transporter activity (GO:0022857) that are differentially expressed between BSCs and MCs irrespective of fold change.

Table S3. K^+^ channel genes differentially expressed between BSCs and MCs.

Data S1. Excel file of 90 genes differentially expressed between BSCs and MCs.

Protocols S1. Collection of single protoplasts for RNA extraction.

Protocols S2. Protoplast pooling for RNA extraction.

Protocols S3. Normalization genes and primers for qRT-PCR.

Protocols S4. Patch-clamp data analysis: LJP corrections and Boltzmann fitting.

Protocols S5. Ratiometric evaluation of protoplast membrane potential.

Movie S1. Collection of single leaf protoplasts using a homemade plastic suction pipette.

## Data Deposition

Raw microarray data. Gene Expression Omnibus (GEO). Accession number GSE85463.

https://www.ncbi.nlm.nih.gov/geo/query/acc.cgi?acc=GSE85463

## Supplementary Material

supplementary_Figures_S1_S8

supplementary_Tables_S1_S3

supplementary_Dataset_S1

supplementary_movie_S1

supplementary_Protocols

## Abbreviations:

ABA: abscisic acid
APX2: ascorbate peroxidase
BS: bundle sheath
BSC: bundle sheath cell
E_H_: holding potential
E_K_: K^+^ equilibrium potential
E_M_: membrane potential
E_rev_: reversal potential
G: membrane conductance
G’: specific conductance
GFP: green fluorescent protein
GO: gene ontology
I-V: current-voltage
LJP: liquid junction potential
MC: mesophyll cell
NREL: normalized raw expression level
P_O_: open channels
Rs: series resistance
SCR: scarecrow
TGs: transporter genes
qRT-PCR: quantitative real-time PCR.
