# Supplementary material for: Differential gene expression and transport functionality in the bundle sheath versus mesophyll – a potential role in leaf mineral homeostasis
Source: J Exp Bot. 2017 Apr 12;68(12):3179–90. doi: 10.1093/jxb/erx067 (PMC5853479; doi:10.1093/jxb/erx067)
Supplement: supplementary_Figures_S1_S8 [file erx067_suppl_supplementary_figures_s1_s8.pdf]

A

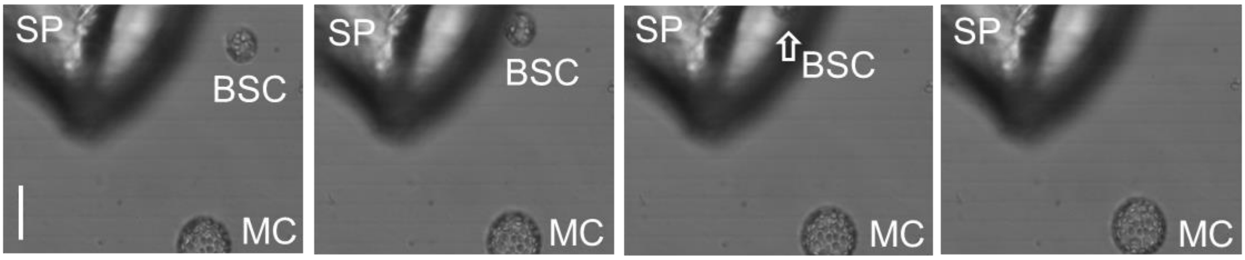

B

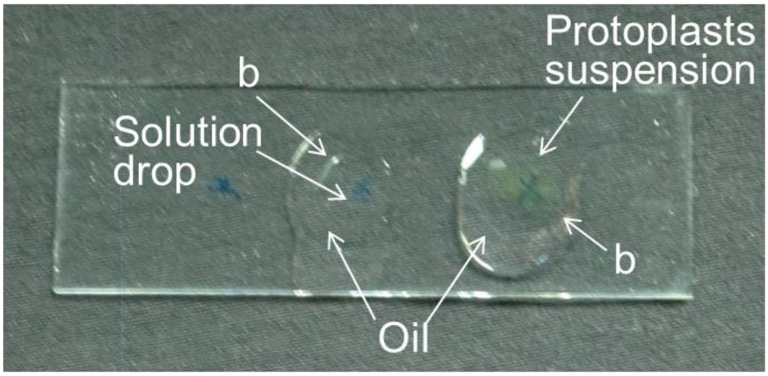

FIGURE S1. Manual collection of individual protoplasts for expression profiling. **A.** Time-lapse (left to right) monochromatic images of a bundle sheath cell protoplast (BSC) progressing towards the opening of the suction pipette (SP). Note the mesophyll cell protoplast (MC) which did not enter the pipette. The purity of the collection was ensured by visual discrimination between the GFP-fluorescing BSCs protoplasts and the non-fluorescing MCs protoplasts (see also Movie S1). Scale bar 50 μm. **B.** A microscope slide with two Xs marked on the bottom (in blue), to assist in pipette navigation. On the right, a 20 μL leaf protoplasts suspension covered by mineral oil. Note the green hue of the protoplasts. This is the source for protoplasts aspiration. On the left, a clear 5 μL droplet of wash solution covered by mineral oil prepared for the validation, i.e., the release of the aspirated protoplast, reexamination and then collection into an Eppendorf tube. Note the boundaries (b) of the solution drops within the oil drops.

A

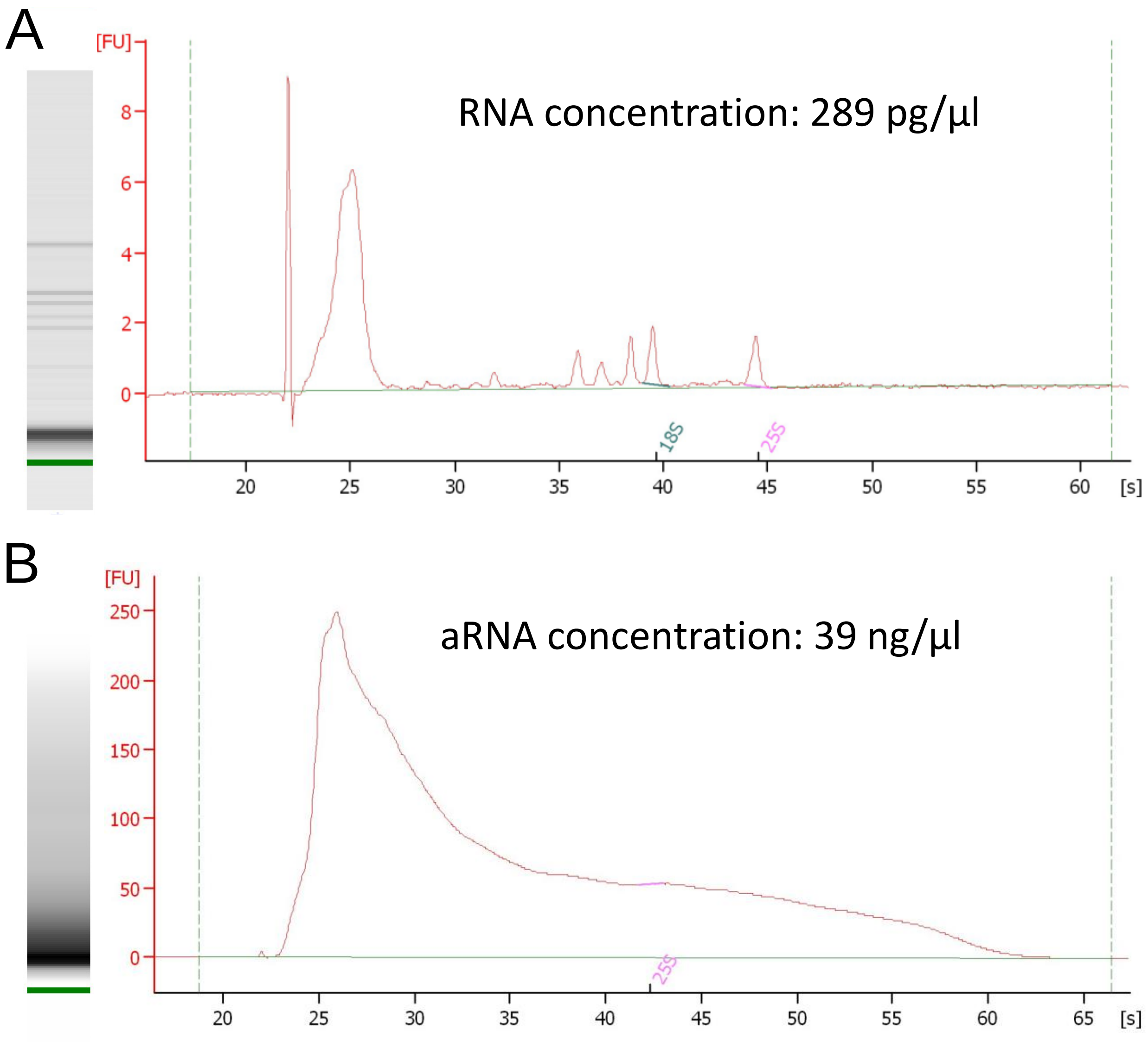

FIGURE S2. BioAnalyser RNA integrity test: representative results**.** Samples taken from Electrophoresis File Run Summary of assayed Pico Plant RNA. **A:** RNA from 20 MCs, isolated using ExpressArt® LCM RNAready isolation kit. Note the distinct bands indicating normal intact plant RNA. **B**: Twice-amplified RNA of MCs (aRNA). No bands are expected after this procedure.

Supplementary Fig. S2, Wigoda et al., 2017

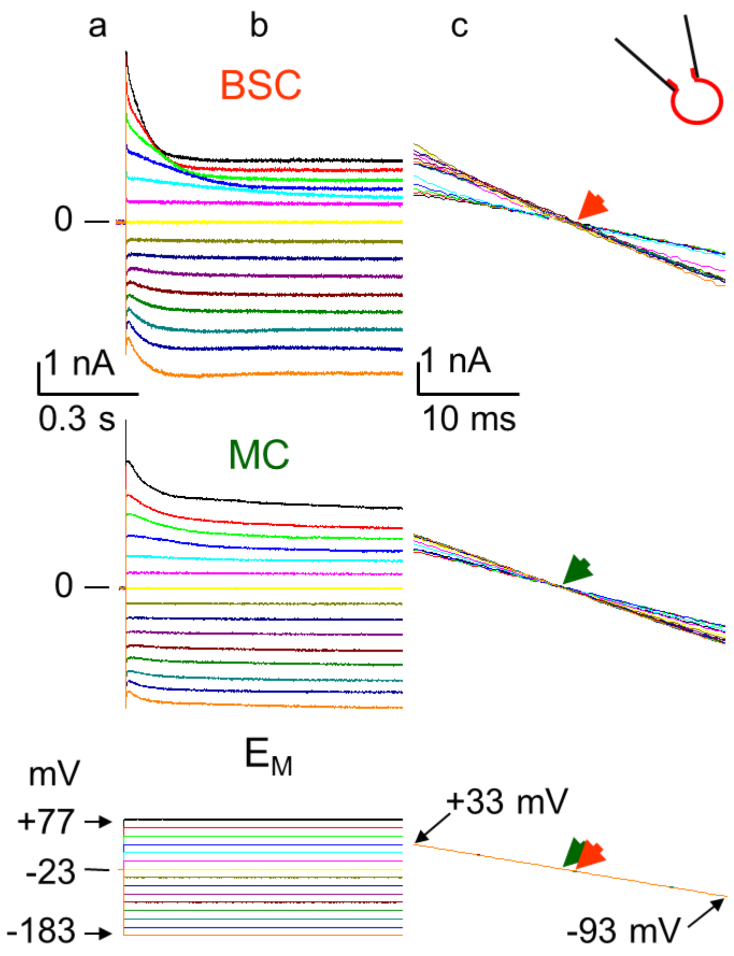

FIGURE S3. Time- and voltage-dependent whole-cell currents from a BSC and an MC (exemplary traces). The current traces are superimposed and color-coded the same as the corresponding voltage pulses below (E_M).

(a-c) indicate the chief components (along vertical panels) of the voltage sweeps and the corresponding current traces: (a) the holding potential and current, (b) the pre-pulses and elicited currents, (c) the "G-V-testing" voltage ramp and current ramps. Inward time-dependent currents (in panel b) appear as downward deflections below the null current level (0 in panel a). Inset: the recording configuration. Arrowheads: the crossover points of the current-ramps (orange: BSC, green: MC). Note the different time bases of the square pre-pulse and the subsequent G-V-testing ramp (three ms of capacitative transients at the ramp onset have been blanked out for visual clarity); the G-V-testing ramp was elicited immediately after the square pulse. Prior to and between the consecutive pulse sets ("sweeps"), the membrane potential was held at -23 mV for 20-30s.

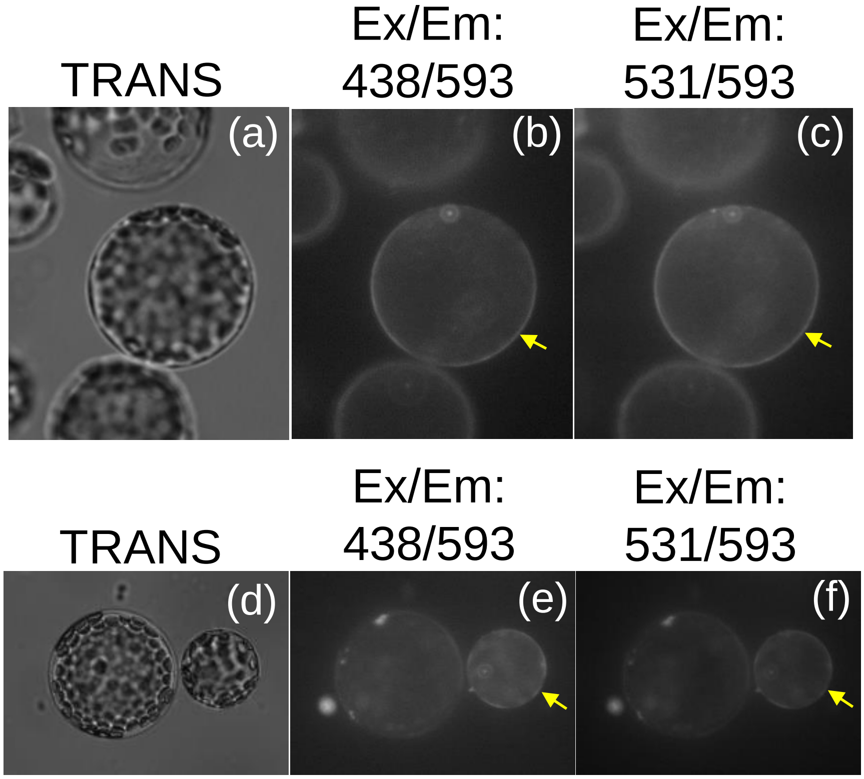

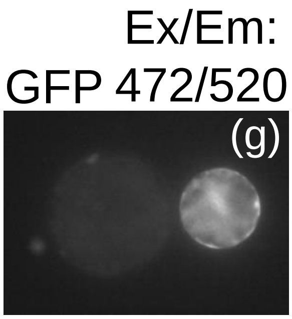

FIGURE S4. Images of Arabidopsis leaf protoplasts stained with the potentiometric membrane dye di-8-ANEPPS. **(a-c)** Images of MCs; (**d-g**) images of a BSC (small), and an MC (large), (**a, d**) Images in TRANSmitted light, (**b, e**) and (**c, f**) di-8-ANEPPS fluorescence at the indicated excitation/emission (Ex)/Em) wavelengths, in nm. Note the bright circumference (membrane fluorescence) of the protoplast in focus (arrows). The brightest-fluorescing regions of the membrane were selected using ImageJ (see Supplementary Protocols S5). The diameter of an MC: approx. 50 μm, of a BSC: approx. 25 μm, (**g**) GFP fluorescence image acquired at the specified Ex/Em wavelengths, in nm, of the same field of vision as in d-f. Note the GFP-fluorescence of the BSC. Note also the *lack* of correspondence between the fluorescence intensities of the GFP and the di-8-ANEPPS images of the same cells. More details in Supplementary Protocols S5.

A

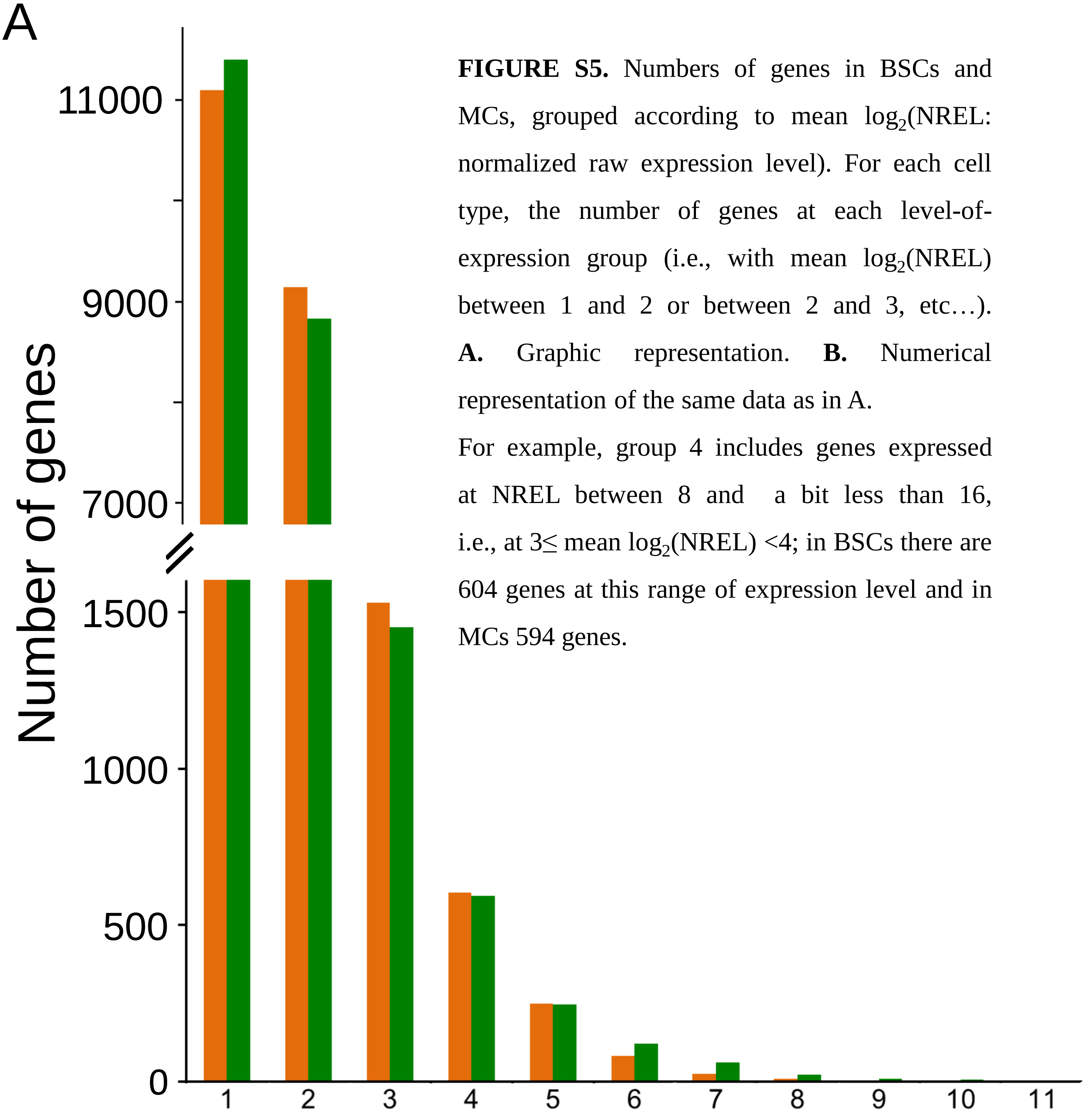

FIGURE S5. Numbers of genes in BSCs and MCs, grouped according to mean log_2(NREL: normalized raw expression level). For each cell type, the number of genes at each level-of-expression group (i.e., with mean log_2(NREL) between 1 and 2 or between 2 and 3, etc…). **A.** Graphic representation. **B.** Numerical representation of the same data as in A.

For example, group 4 includes genes expressed at NREL between 8 and a bit less than 16, i.e., at $3 \leq$ mean log_2(NREL) <4; in BSCs there are 604 genes at this range of expression level and in MCs 594 genes.

B

	1	2	3	4	5	6	7	8	9	10	11
■ BSCs	11100	9144	1529	604	250	82	25	11	0	1	0
■ MCs	11399	8830	1450	594	246	123	63	23	10	7	1

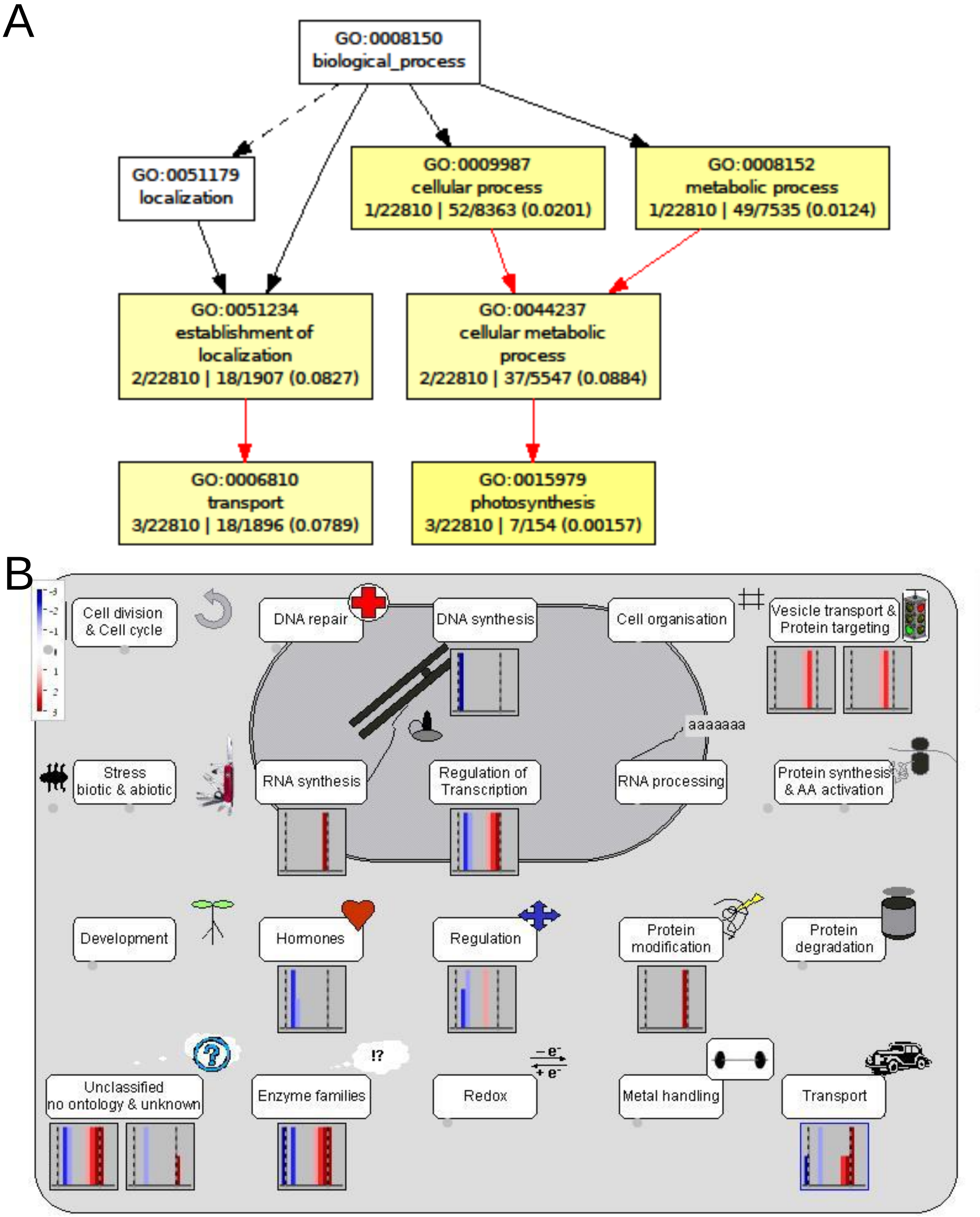

FIGURE S6. Ontology analysis of genes showing differential expression in BSCs vs. MCs, using GOEAST and MapMan (*Continued on next page*).

Supplementary Fig. S6, Wigoda et al., 2017

FIGURE S6. Ontology Analysis of Genes showing differential expression in BSCs vs. MCs, using GOEAST and MapMan. GO analysis of the 90 genes that showed differential expression in BCs vs. MCs ($P < 0.05$; absolute fold-change ≥ 1.5), **A.** using GOEAST (the darker the box-color - the higher the enrichment), **B.** using MapMan (the enrichment levels are displayed onto a diagram of cell functions overview); note the genes identified in transport functionality.

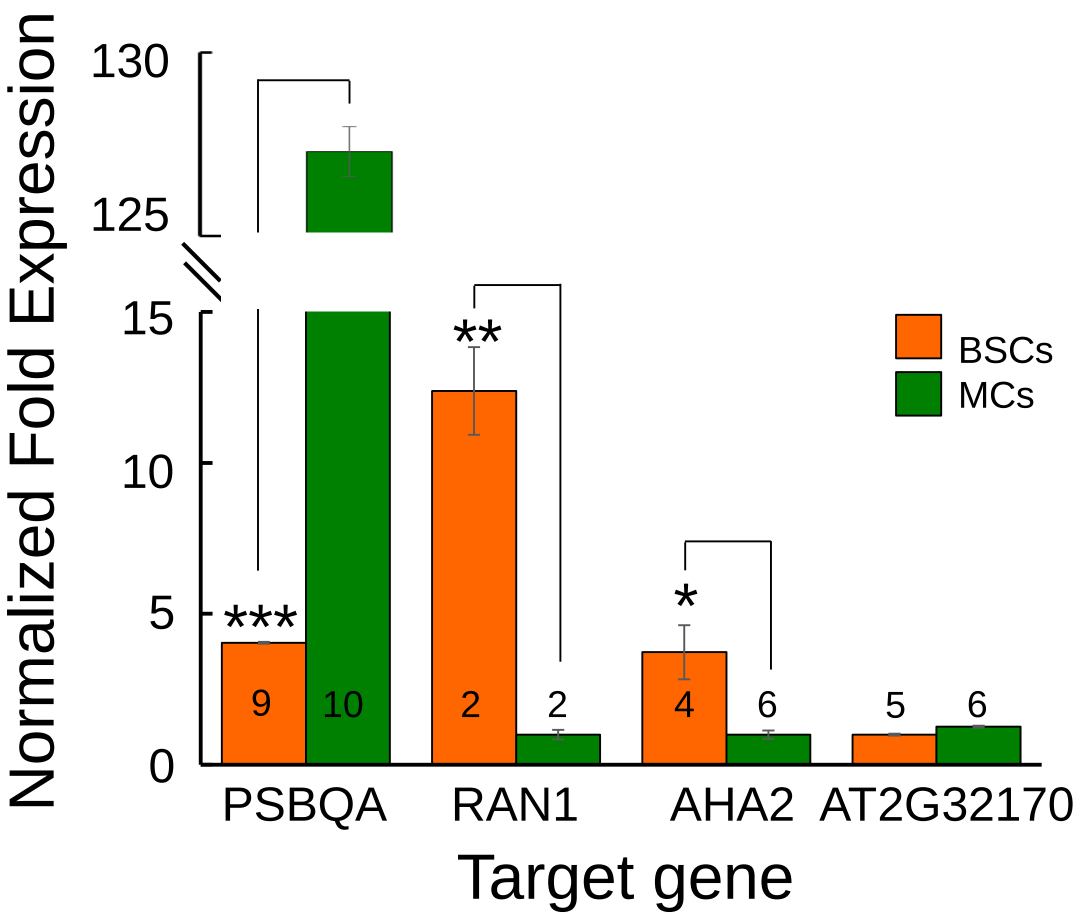

FIGURE S7. qRT-PCR validation of expression in BSCs and MCs. qRT-PCR-determined expression of PSBQA (AT4G21280), RAN1 (AT5G44790), AHA2 (AT4G30190) and AT2G32170, and normalized to the expression of the genes AT5G12240 and AT2G07734. Means (±SE), of the indicated number of samples. Separate comparisons for each gene by Student's t test. Asterisks: the significance level of the differences between the cell types; *: $P < 0.02$, **: $P < 0.01$, ***: $P < 0.001$.

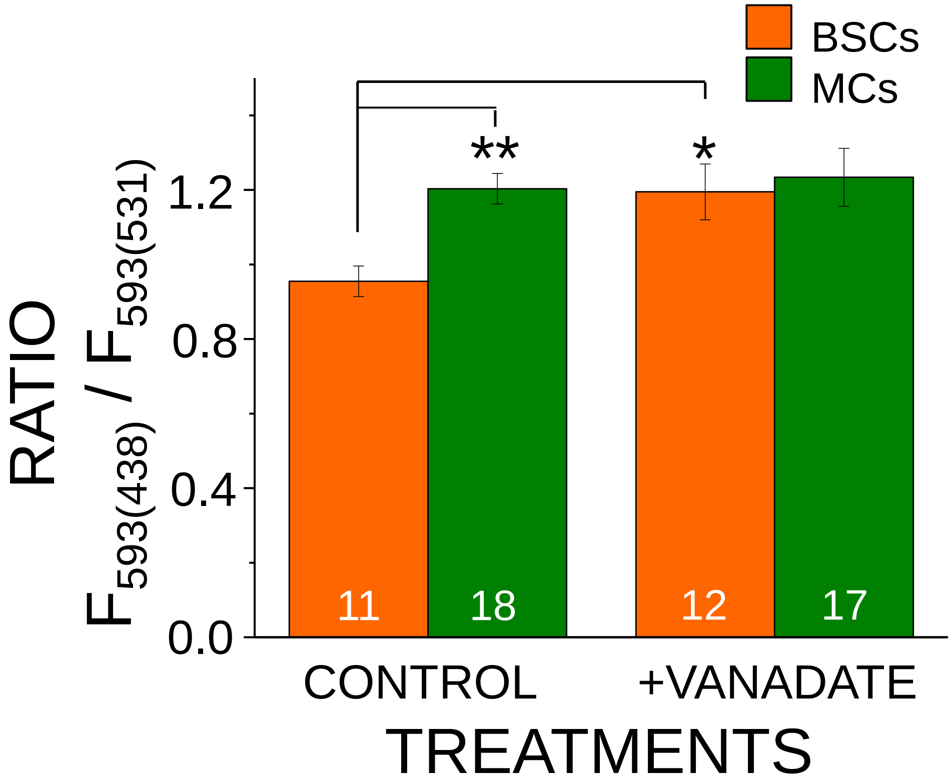

FIGURE S8. The effect of depolarizing agents on the fluorescence ratio of BSCs and MCs protoplasts stained with the potentiometric probe, di-8-ANEPPS. Control: Shown are the mean (±SE) values of ratio from the indicated number of protoplasts for each cell type and treatment in two experiments. +Vanadate: the same experiments as in Control, except the wash solution and the bathing medium during the imaging experiments contained additionally the depolarizing agents 1 mM vanadate and 5 mM KNO_3. Asterisks: significance level of the difference from the ratio of the control BSCs; *****: P<0.02, ******: P<0.001.
