# Supplementary material for: Differential gene expression and transport functionality in the bundle sheath versus mesophyll – a potential role in leaf mineral homeostasis
Source: J Exp Bot. 2017 Apr 12;68(12):3179–90. doi: 10.1093/jxb/erx067 (PMC5853479; doi:10.1093/jxb/erx067)
Supplement: supplementary_Tables_S1_S3 [file erx067_suppl_supplementary_tables_s1_s3.pdf]

Supplementary Tables

Supplementary Table S1. Genes expressed differentially in BSCs vs. MCs, associated with GO:0005215~ transporter activity (with absolute fold-change≥1.5 and P-value <0.05).

The letters in parentheses denote the subcellular localization of the protein (C: chloroplast, EM: endomembranes, G: Golgi, M: mitochondrion, N: nucleus, PM: plasma membrane, U: undetermined localization); --- : marks non-annotated (i.e., nameless) genes, the descriptions are from TAIR; NREL: normalized raw expression level; Note: PDE318 has been added to the list manually (based on TAIR).

TAIR ID	Gene symbol and description	Fold-change	P-value	log_2(NREL)	
				BSCs	MCs
AT1G80770[B]	PDE318<br>ferrous iron transmembrane transporter (U)	4.00	0.020	4.95	2.94
AT4G30190[C]	AHA2<br>hydrogen-exporting ATPase (PM)	3.15	0.032	5.46	3.81
AT5G17850[B]	---<br>Calcium: sodium antiporter activity (EM)	3.03	0.039	5.07	3.47
AT5G01340	--- mSFC1<br>Mitochondrial succinate-fumarate carrier 1 (EM, M)	2.51	0.032	3.44	2.11
AT5G44790[B]	RAN1 ATP dependent copper transporter,<br>vital for ethylene response pathway (EM,U)	1.99	0.017	3.52	2.52
AT1G20350[C]	ATTIM17-1<br>Mitochondrial inner membrane translocase (EM)	1.76	0.016	2.90	2.09
AT5G17020	XPO1A<br>alternative oxidase 1A (EM, N)	1.56	0.023	2.74	2.10
AT2G28520	VHA-A1 (Vacuolar proton ATPase A 1); (EM, V)	-1.56	0.049	2.13	2.78
AT2G47490	---- NDT1<br>chloroplast-localized NAD^+ transporter (EM,C)	-1.63	0.044	2.49	3.19
AT2G31530	EMB2289 (EMBRYO DEFECTIVE 2289) or SCY2; P-P-bond-hydrolysis-driven protein transmembrane (EM,C)	-1.72	0.048	2.07	2.85
AT2G38410	---<br>protein transporter activity (G, PM)	-1.89	0.037	2.30	3.21
AT5G15090[A]	VDAC3<br>voltage dependent anion channel 3 (EM, PM?)	-3.83	0.007	2.60	4.54

Supplementary Table S2. 46 Genes expressed differentially in BSCs vs. MCs (irrespective of fold change) associated with GO:0022857~transmembrane transporter activity.

The letters in parentheses denote the subcellular localization of the protein (C: chloroplast, EM: endomembranes, G: Golgi, M: mitochondrion, N: nucleus, PM: plasma membrane, U: undetermined localization); --- marks non-annotated (i.e., nameless) genes, the descriptions are from TAIR; NREL: normalized raw expression level; Superscripts **A- C** denote the sub-groups of the 46 transporter genes shared with the other categories (shown in Venn diagram in Figure 3c), and identified and described in the text; Note: AT5G17020, AT2G47490 and AT2G38410 were added to the list manually (based on DAVID).

**TAIR ID**	**Gene symbol and description**	**Fold-change**	**P-value**	**log_2(NREL)**	
				**BSCs**	**MCs**
AT1G80770**B**	PDE318<br>ferrous iron transmembrane transporter (U)	4.00	0.020	4.95	2.94
AT4G30190**C**	AHA2<br>hydrogen-exporting ATPase (PM)	3.15	0.032	5.46	3.81
AT5G17850**B**	---<br>Calcium: sodium antiporter activity (EM)	3.03	0.039	5.07	3.47
AT5G01340	--- mSFC1<br>Mitochondrial succinate-fumarate carrier 1 (EM, M)	2.51	0.032	3.44	2.11
AT5G44790**B**	RAN1 ATP dependent copper transporter, vital for ethylene response pathway (EM,U)	1.99	0.017	3.52	2.52
AT1G20350**C**	ATTIM17-1<br>Mitochondrial inner membrane translocase (EM)	1.76	0.016	2.90	2.09
AT5G17020	XPO1A<br>alternative oxidase 1A (EM, N)	1.56	0.023	2.74	2.10
AT5G19500**B**	--- putative amino acid transporter that localizes to the chloroplast inner envelope membrane (C)	1.44	0.010	3.88	3.35
AT5G05310**B**	--- TLC ATP/ADP transporter	1.36	0.013	3.59	3.15
AT3G23550	--- MATE (multi antimicrobial extrusion) efflux family protein	1.25	0.003	2.21	1.90
AT1G80050	APT2<br>adenine phosphoribosyl transferase 2	1.23	0.029	2.25	1.95
AT5G13750	ZIFL1 (ZINC INDUCED FACILITATOR-like 1); tetracycline:hydrogen antiporter	1.22	0.021	2.20	1.91
AT1G51500	CER5 (ECERIFERUM 5); ATPase, coupled to transmembrane movement of substances	1.20	0.012	2.43	2.16

Supplementary Table S2, *Cont.*					
TAIR ID	Gene symbol and description	Fold-change	P-value	log_2(NREL)	
				BSCs	MCs
AT2G14670	ATSUC8 (Sucrose-proton symporter 8); carbohydrate transmembrane transporter	1.18	0.012	1.94	1.71
AT4G10380	NIP5;1; arsenite transmembrane transporter, boron/water channel	1.15	0.017	2.00	1.79
AT2G17470	--- ALMT6, a member of the aluminum-activated malate transporter family	1.15	0.050	2.08	1.88
AT3G28380	PGP17 (P-GLYCOPROTEIN 17); ATP binding / ATPase	1.15	0.049	1.67	1.48
AT5G37060	CHX24 sodium:hydrogen antiporter	1.13	0.044	1.55	1.37
AT1G53390	--- ATP-BINDING CASSETTE G24 (ABCG24)	1.12	0.039	2.47	2.30
AT1G71870	--- MATE (multi antimicrobial extrusion) efflux family protein	1.12	0.006	1.87	1.70
AT2G40540	KT2 (POTASSIUM TRANSPORTER 2); potassium ion transmembrane transporter	1.11	0.013	1.94	1.79
AT5G62160	AtZIP12 (ZINC TRANSPORTER 12 PRECURSOR); cation transmembrane transporter/ metal	1.11	0.041	2.24	2.09
AT3G51480	ATGLR3.6 (GLUTAMATE RECEPTOR 3.6); intracellular ligand-gated ion channel	1.10	0.010	2.11	1.96
AT2G19110	HMA4; cadmium ion transmembrane transporter/ cadmium-transporting ATPase/ zinc	1.10	0.018	1.79	1.65
AT2G35060	KUP11; potassium ion transmembrane transporter	1.10	0.048	1.80	1.66
AT5G03570	ATIREG2 (IRON-REGULATED PROTEIN 2); nickel ion transmembrane transporter	1.09	0.011	1.85	1.73
AT1G06970	CHX14 (CATION/HYDROGEN EXCHANGER 14); monovalent cation:proton antiporter/ sodium	1.07	0.035	1.79	1.69
AT3G13100	ATMRP7; ATPase, coupled to transmembrane movement of substances	1.07	0.030	1.51	1.41
AT2G30080	ZIP6; cation transmembrane transporter/ metal ion transmembrane transporter	-1.04	0.044	1.97	2.03
AT2G01770	VIT1 (vacuolar iron transporter 1); iron ion transmembrane transporter	-1.06	0.046	1.58	1.66
AT1G12480	OZS1 (OZONE-SENSITIVE 1) /SLAC1 anion channel	-1.07	0.007	2.02	2.12
AT5G50790	--- sweet10 sucrose efflux transporter	-1.09	0.010	2.24	2.36
AT5G11690	ATTIM17-3; P-P-bond-hydrolysis-driven protein transmembrane transporter/ protein	-1.11	0.034	4.07	4.23
AT4G30360	CNGC17 cyclic nucleotide binding / ion channel	-1.13	0.009	1.42	1.61
AT2G07560	AHA6 (Arabidopsis H(+)-ATPase 6); ATPase	-1.14	0.049	2.08	2.27

**Supplementary Table S2**, *Cont.*					
**TAIR ID**	**Gene Symbol & description**	**Fold change**	**p-value**	**log_2(NREL)**	
				**BSCs**	**MCs**
AT3G60330	AHA7 (Arabidopsis H(+)-ATPase 7); hydrogen-exporting ATPase, phosphorylative mechanism	-1.14	0.025	1.61	1.80
AT4G01010	ATCNGC13; calmodulin binding / cyclic nucleotide binding / ion channel	-1.15	0.027	2.02	2.23
AT3G03620	--- MATE (multi antimicrobial extrusion) efflux family protein	-1.16	0.009	1.80	2.01
AT2G04620	--- Cation efflux transporter	-1.18	0.024	1.79	2.02
AT2G13650	GONST1 (Golgi Nucleotide Sugar Transporter 1); GDP-mannose transmembrane transporter	-1.19	0.004	2.04	2.29
AT5G57490	VDAC4 voltage-gated anion channel 4	-1.20	0.048	1.60	1.86
AT2G28520	VHA-A1 (Vacuolar proton ATPase A 1); (EM, V)	-1.56	0.049	2.13	2.78
AT2G47490	---- NDT1 chloroplast-localized NAD^+ transporter (EM,C)	-1.63	0.044	2.49	3.19
AT2G31530	EMB2289 (EMBRYO DEFECTIVE 2289); P-P-bond-hydrolysis-driven protein transmembrane (EM,C)	-1.72	0.048	2.07	2.85
AT2G38410	--- protein transporter activity (G, PM)	-1.89	0.037	2.30	3.21
AT5G15090[A]	VDAC3 Voltage dependent anion channel 3 (EM, PM?)	-3.83	0.007	2.60	4.54

Table S3. Genes of K^+ channels in BSCs vs. MCs.

NREL: normalized raw expression level.

TAIR ID	Gene Symbol & description	Fold change	p-value	log_2(NREL)	
				BSCs	MCs
AT4G18290	**KAT2** (POTASSIUM CHANNEL IN ARABIDOPSIS THALIANA 2); cyclic nucleotide binding / inward	1.13	0.45	1.86	1.68
AT4G32650	**ATKC1** (ARABIDOPSIS THALIANA K^+ RECTIFYING CHANNEL 1); cyclic nucleotide binding / inward	1.10	0.67	1.77	1.66
AT4G22200	**AKT2/3** (ARABIDOPSIS POTASSIUM TRANSPORT 2/3); cyclic nucleotide binding / inward rectifier	1.06	0.26	1.92	1.78
AT5G46370	**KCO2** (CA^{2+} ACTIVATED OUTWARD RECTIFYING K^+ CHANNEL 2); calcium ion binding / outward rectifier	1.06	0.83	2.01	1.95
AT2G25600	**SPIK** (Shaker Pollen Inward K^+ channel); cyclic nucleotide binding / inward rectifier potassium	1.04	0.50	1.93	1.86
AT4G01840	**KCO5** (CA^{2+} ACTIVATED OUTWARD RECTIFYING K^+ CHANNEL 5); outward rectifier potassium channel	1.04	0.65	1.72	1.70
AT5G55630	**ATKCO1**; calcium-activated potassium channel/ ion channel/ outward rectifier potassium channel	1.02	0.96	2.09	2.16
AT2G26650	**AKT1** (ARABIDOPSIS K TRANSPORTER 1); cyclic nucleotide binding / inward rectifier potassium	-1.00	0.99	2.69	2.53
AT5G46240	**KAT1** (POTASSIUM CHANNEL IN ARABIDOPSIS THALIANA 1); cyclic nucleotide binding / inward	-1.00	0.97	1.53	1.53
AT4G32500	**AKT5**; cyclic nucleotide binding / inward rectifier potassium channel	-1.04	0.61	1.86	1.94
AT4G18160	**KCO6**; outward rectifier potassium channel	-1.04	0.83	2.48	2.63
AT3G02850	**SKOR**; cyclic nucleotide binding / inward rectifier potassium channel/ outward rectifier	-1.07	0.82	2.20	2.29
AT5G37500	**GORK** (GATED OUTWARDLY-RECTIFYING K^+ CHANNEL); cyclic nucleotide binding / inward rectifier	-1.08	0.42	1.71	1.79
AT5G46360	**KCO3** (CA^{2+} ACTIVATED OUTWARD RECTIFYING K^+ CHANNEL 3); calcium ion binding / outward rectifier	-1.15	0.09	1.73	1.75
AT1G02510 *	**TPK4 / KCO4**				

* No data in our experiment.
