# Supplementary material for: Differential gene expression and transport functionality in the bundle sheath versus mesophyll – a potential role in leaf mineral homeostasis
Source: J Exp Bot. 2017 Apr 12;68(12):3179–90. doi: 10.1093/jxb/erx067 (PMC5853479; doi:10.1093/jxb/erx067)
Supplement: supplementary_Protocols [file erx067_suppl_supplementary_protocols.pdf]

Journal of Experimental Botany

Differential gene expression and transport functionality in the bundle sheath versus mesophyll – a potential role in leaf mineral homeostasis

Noa Wigoda, Metsada Pasmanik-Chor, Tianyuan Yang, Ling Yu, Menachem Moshelion and Nava Moran

Supplementary Protocols

Supplementary Protocols S1. Collection of single type protoplasts for RNA extraction

Protoplasts were isolated as described by (Shatil-Cohen *et al.*, 2011;Shatil-Cohen *et al.*, 2014). For GFP excitation we used an inverted epifluorescence microscope Eclipse TS100 (Nikon, http://www.nikoninstruments.com) equipped with a Philips LED lamp 485±5 nm and an emission filterset GFP 3035B BrightLine 520±18 nm from Semrock (http://www.semrock.com). Leaf protoplasts were placed on a microscope slide in 20 µL suspension droplets covered by mineral oil (Sigma M-5904) (Supplementary Fig. S1B). Individual bundle sheath protoplasts (with a diameter of 25-30 µm) were identified by their GFP fluorescence and the mesophyll protoplasts (with a diameter of 40-45 µm) – by their lack of fluorescence (Supplementary Fig. S2). Protoplasts of these sizes were used in experiments by Shatil-Cohen et al., 2011. The identified protoplasts were collected manually from the 20 µL droplet, one by one, under a bright-field illumination, using a 20X objective. 1 to 5 protoplasts were aspirated and then released into a second 5 µL droplet of wash solution covered by mineral oil using a home-made plastic micro suction-pipette, with a tip aperture of 200 µm (Supplementary Fig. S1A mounted on a micromanipulator and connected to a micrometer syringe (GILMONT® Instruments). After visual validation of the purity of the collection in the 2nd droplet (fluorescent bundle sheath protoplasts or non-fluorescent mesophyll protoplasts), single-type protoplasts were transferred using the suction-pipette (demonstrated in a 7.5 sec. long Movie S1, filmed at 4 Hz) into a 1.5 ml Eppendorff tube and flash-frozen in liquid nitrogen. The frozen samples were kept at -80ºC until further analysis.

Supplementary Protocols S2. Protoplasts pooling for RNA extraction

The protoplasts pooling was performed as follows: first, the kit lysis buffer (without N-Carrier) was used to rinse an Eppendorff tube with a frozen single-type protoplast (or protoplasts) by gentle pipetting. Then, the lysate (whole contents of the tube) was transferred to the next tube with another frozen protoplast of the same type and the rinse procedure repeated, and so on, until all 20 cells were pooled together. Finally, all the tubes were spun and remaining lysate was added to the pool. The tubes were kept on ice during the pooling. The rest of the extraction procedure was according to the manufacturer's protocol.

Supplementary Protocols S3. Normalization genes and primers for real-time PCR

Transcripts of stably expressed genes constitute crucial internal references for normalization of gene expression data (Czechowski *et al*., 2005). Therefore, we choose for normalization genes with stable expression in our sample types, both in the microarray and in RT-PCR analyses (AT5G12240 [Octanol transferase], similarly to Czechowski et al. (2005) and AT2G07734 [a ribosomal protein S4]).

Primers for AT5G12240 and AT2G32170 were as described (by Czechowski *et al.* 2005). AHA2 primers were as described (by Haruta *et al*., 2010). In addition, the following primers were used: for AT2G07734: AT2G07734-F (5'-TGG GCG GAT AAA TTC GGG TTC C-3') and AT2G07734-R (5'-AGC GGA CTT TCT GAC TTC AAC TGG-3'), for PSBQA (AT4G21280): PSBQA-F (5'- GTT TTA CTT ACA GCC ATT GCC -3') and PSBQA-R (5'- CAT AAC GAA GAT AAG AAG CCT TG -3'), and for RAN1 AT5G44790): RAN1-F (5'- CTT ACT TCT ACT CTG TTG GGG-3'), and RAN1-R (5'- CCT TCA GTT AGT AAA ATC GCT GT-3').

Supplementary Protocols S4. Patch-clamp data analysis: LJP corrections and Boltzmann fitting.

Liquid Junction Potential (LJP) correction. Based on the composition of the internal and external solutions, the values of the LJP, were calculated using the pClamp 9.2 suite calculator and added to all values of E_M. Thus, for the pair of "internal" and "low K" bath solutions, LJP was -23 mV and with 30K bath solution (and the same "internal" solution) LJP was -14 mV (the list of solutions is detailed in Experimental procedures).

Analysis of voltage dependence of membrane conductance. The specific membrane conductance, G', was calculated from the membrane conductance, G (obtained, in turn, from the linear slopes of the current and voltage ramps converted to current-voltage relationships), according to Eq. S1, where r is the radius of the globular protoplast, in cm:

Eq. S1. $$G' = \frac{G}{4\times \pi \times r^2}$$

Individual G'-E_M relationships were fitted with the Boltzmann equation (Hille, 2001), using Origin (Ver. 7, Origin Lab Co., Northampton, MA), according to Eq. S2:

Eq. S2. $$G' = G'_b + \frac{G'_{max} - G'_b}{1+ e^{-zF(E_M - E_{1/2})/RT}}$$

Where G'_b is the basal (background) conductance, G'_{max} is the maximum attainable conductance, and thus $G'_{max\_net} = G'_{max}-G'_b$ is the maximum attainable conductance above G'_b, E_M is the membrane potential, $E_{1/2}$ is the E_M at which half of G'_{max_net} is attained, z is the effective charge of the gating subunit, F is Faraday's constant, R is the universal gas constant, and T is the absolute temperature. The individual best-fit parameters values obtained from the fit were then averaged separately for each cell type.

Channel open probability, P_O, was calculated by Eq. S3 arrived at by subtracting G'_b from G' and dividing by G'_{max_net}:

Eq. S3. $$Po = \frac{1}{1+ e^{-zF(E_M - E_{1/2})/RT}}$$

Supplementary Protocols S5. Ratiometric evaluation of protoplasts membrane potential

The ANEPPS membrane dyes (di-4 ANEPPS and di-8-ANEPPS) have been used in various animal cells to evaluate the transmembrane potential (E_M) (Montana *et al.*, 1989;Pucihar *et al.*, 2009;Zhang *et al.*, 1998), but only rarely in plants (e.g., in pollen tube protoplasts, Breygina et al., 2009). Zhang et al. validated the use of ratiometric imaging of di-8-ANEPPS fluorescence by calibrating these measurements in neuronal cells with concomitant E_M changes imposed by patch clamp. Ratiometric measurement using fluorescent dyes yield data insensitive to dye leakage or to photo-

bleaching and permit relatively reliable steady-state measurements, which is the type of measurements that we performed.

Image acquisition. Images were acquired using the CellR software (Olympus), via a U-APO 40X water-immersion objective. Bandpass fluorescence filters (from Semrock, https://www.semrock.com) were used for the dual excitation of ANEPPS and fluorescence recording at a single emission wavelength: blue exciter: 438±12 nm, green exciter: 531±20 nm and a red emitter: 593±20 nm (with a dichroic 562 nm long pass filter). For GFP, the GFP-3035B-000 set from Semrock was used: (exciter: 472±15 nm, emitter: 520±18 nm).

Image analysis. The fluorescence images were processed using ImageJ with the Ratio Plus plugin (NIH, USA freeware, see http://rsbweb.nih.gov/ij/; Supplementary Fig. S4). ImageJ is a public domain software (Rasband, W.S., ImageJ, U. S. National Institutes of Health, Bethesda, Maryland, USA, http://imagej.nih.gov/ij/, 1997-2015).

Mean background fluorescence intensity, determined from an empty area outside the protoplast, was subtracted from the image. The brightest membrane areas selected using ImageJ for ratio calculation had a mean intensity about 10% higher than that of the background. Pixel-by-pixel ratio between the two fluorescence images was calculated by 'RatioPlus' (an ImageJ plugin) and the mean ratio values of the selected membrane area of each protoplast were averaged for each cell type (Fig. 5 and Supplementary Fig. S8).

References for Supplementary Data

Czechowski T, Stitt M, Altmann T, Udvardi MK & Scheible W-R. 2005. Genome-Wide Identification and Testing of Superior Reference Genes for Transcript Normalization in Arabidopsis. *Plant Physiology* 139, 5-17.

Haruta M, Burch HL, Nelson RB, Barrett-Wilt G, Kline KG, Mohsin SB, Young JC, Otegui MS & Sussman MR. 2010. Molecular Characterization of Mutant Arabidopsis Plants with Reduced Plasma Membrane Proton Pump Activity. *Journal of Biological Chemistry* 285, 17918-17929.

Hille B. 2001. *Ion Channels of Excitable Membranes*. Third Edition edn. Sinauer Associates, Inc.

Montana V, Farkas DL & Loew LM. 1989. Dual-wavelength ratiometric fluorescence measurements of membrane potential. *Biochemistry* 28, 4536-4539.

Pucihar G, Kotnik T & Miklavčič D. 2009. Measuring the Induced Membrane Voltage with Di-8-ANEPPS. *J Vis Exp*, 1659.

Shatil-Cohen A, Attia Z & Moshelion M. 2011. Bundle-sheath cell regulation of xylem-mesophyll water transport via aquaporins under drought stress: a target of xylem-borne ABA? *The Plant Journal* 67, 72-80.

Shatil-Cohen A, Sibony H, Draye X, Chaumont F, Moran N & Moshelion M. 2014. Measuring the osmotic water permeability coefficient (Pf) of spherical cells: isolated plant protoplasts as an example. *J Vis Exp*, e51652.

Zhang J, Davidson RM, Wei MD & Loew LM. 1998. Membrane electric properties by combined patch clamp and fluorescence ratio imaging in single neurons. *Biophys J* 74, 48-53.
